# Sessile snails, dynamic genomes: gene rearrangements within the mitochondrial genome of a family of caenogastropod molluscs

**DOI:** 10.1186/1471-2164-11-440

**Published:** 2010-07-19

**Authors:** Timothy A Rawlings, Martin J MacInnis, Rüdiger Bieler, Jeffrey L Boore, Timothy M Collins

**Affiliations:** 1 Cape Breton University, 1250 Grand Lake Road, Sydney, NS B1P 6L2, CANADA; 2 University of British Columbia, 2239 West Mall, Vancouver, BC V6T 1Z4, CANADA; 3 Field Museum of Natural History, 1400 S. Lake Shore Dr, Chicago, IL 60605-2496, USA; 4 Genome Project Solutions, Inc.,1024 Promenade Street, Hercules, CA 94547, USA; 5 Florida International University, 11200 SW 8th Street, University Park, Miami, FL 33199, USA; 6 National Science Foundation, 4201 Wilson Boulevard, Arlington, VA 22230, USA

## Abstract

**Background:**

Widespread sampling of vertebrates, which comprise the majority of published animal mitochondrial genomes, has led to the view that mitochondrial gene rearrangements are relatively rare, and that gene orders are typically stable across major taxonomic groups. In contrast, more limited sampling within the Phylum Mollusca has revealed an unusually high number of gene order arrangements. Here we provide evidence that the lability of the molluscan mitochondrial genome extends to the family level by describing extensive gene order changes that have occurred within the Vermetidae, a family of sessile marine gastropods that radiated from a basal caenogastropod stock during the Cenozoic Era.

**Results:**

Major mitochondrial gene rearrangements have occurred within this family at a scale unexpected for such an evolutionarily young group and unprecedented for any caenogastropod examined to date. We determined the complete mitochondrial genomes of four species (*Dendropoma maximum*, *D. gregarium*, *Eualetes tulipa*, and *Thylacodes squamigerus*) and the partial mitochondrial genomes of two others (*Vermetus erectus *and *Thylaeodus sp*.). Each of the six vermetid gastropods assayed possessed a unique gene order. In addition to the typical mitochondrial genome complement of 37 genes, additional tRNA genes were evident in *D. gregarium *(*trnK*) and *Thylacodes squamigerus *(*trnV*, *trnL_UUR_*). Three pseudogenes and additional tRNAs found within the genome of *Thylacodes squamigerus *provide evidence of a past duplication event in this taxon. Likewise, high sequence similarities between isoaccepting leucine tRNAs in *Thylacodes*, *Eualetes*, and *Thylaeodus *suggest that tRNA remolding has been rife within this family. While vermetids exhibit gene arrangements diagnostic of this family, they also share arrangements with littorinimorph caenogastropods, with which they have been linked based on sperm morphology and primary sequence-based phylogenies.

**Conclusions:**

We have uncovered major changes in gene order within a family of caenogastropod molluscs that are indicative of a highly dynamic mitochondrial genome. Studies of mitochondrial genomes at such low taxonomic levels should help to illuminate the dynamics of gene order change, since the telltale vestiges of gene duplication, translocation, and remolding have not yet been erased entirely. Likewise, gene order characters may improve phylogenetic hypotheses at finer taxonomic levels than once anticipated and aid in investigating the conditions under which sequence-based phylogenies lack resolution or prove misleading.

## Background

Animal mitochondrial (mt) genomes typically consist of a circular molecule of DNA encoding 37 genes (2 rRNA genes, 13 protein-encoding genes, and 22 tRNA genes), the arrangement of which is often highly conserved within major taxonomic groups [1]. Consequently, when gene rearrangements occur, they may provide compelling phylogenetic markers that can corroborate or contradict hypotheses based on primary sequence data and provide resolution for deeper nodes that are often weakly supported in sequence-based phylogenies [2-6]. With recent technological and methodological advances (e.g., rolling circle amplification: [7,8]; next generation sequencing technologies: [9]), and associated decreasing costs of DNA sequencing, the amplification and sequencing of whole mt genomes has become routine. As a result, there has been a marked increase in the sequencing of whole animal mt genomes over the past decade as well as the development of computational methods to extract phylogenetic information from these genomes through inferences of past gene dynamics [10-12]. To date, 1868 complete metazoan mt genomes are available in the NCBI Genomes database http://www.ncbi.nlm.nih.gov/guide/genomes/; January 8, 2010), the majority belonging to arthropods (293) and vertebrates (1292).

Compared to other major metazoan phyla, molluscan mitochondrial genomes are poorly represented at NCBI [13], with only 78 complete mt genomes available as of January, 2010. Despite this, molluscan mt genomes are beginning to challenge the traditional view that mitochondrial gene orders are stable over long periods of evolutionary time [13-16], a view based largely on the heavily sampled and highly conserved mt genomes of vertebrates. Instead, mollusc mt genomes demonstrate substantial heterogeneity in length and "architecture" [16], reflecting differences in gene complement resulting from gene loss or duplication, as well as changes in the position and strand specificity of tRNA genes, protein-encoding genes, and rRNA genes. Changes in gene arrangement within the Mollusca have been so dramatic that representatives of four classes of molluscs (Gastropoda; Bivalvia; Cephalopoda; Scaphopoda) share remarkably few mitochondrial gene boundaries, with gene orders varying extensively even across major lineages of bivalves as well as gastropods [14]. Changes in gene arrangement have also been observed *within *bivalve and gastropod genera, based on changes in position of: 1) tRNAs and an rRNA gene in the oyster, *Crassostrea *[17], and 2) protein encoding and tRNA genes in the vermetid marine gastropod genus, *Dendropoma *[18]. Differences in gene order are also evident between paternally versus maternally inherited mitochondrial genomes of bivalves exhibiting doubly uniparental inheritance [19], including the unionid freshwater bivalve, *Inversidens japanensis *[14], and the marine venerid clam, *Venerupis (Ruditapes) philippinarum *(NCBI, unpublished). Similar intrageneric gene translocations have now been described in 19 of 144 genera in which two or more complete mt genomes have been sequenced [16], including representatives of the Porifera, Platyhelminthes, Nematoda, Mollusca, Arthropoda and Chordata. Thus, growing evidence suggests that mt genomes of many metazoan phyla may be considerably more plastic than originally believed, with the conserved genome architecture of vertebrates reflecting a derived stabilization of the mt genome and not an ancestral feature [16].

The discovery of mt gene order changes at lower taxonomic levels, as found within the Mollusca, is exciting for several reasons. First, gene dynamics involving translocations and inversions of genes offer the promise of new and robust characters that can be used to support phylogenetic hypotheses at the level of families, genera, and species [18]. Given the comparatively low rate of rearrangement and the astronomical number of possible gene arrangements, convergence is likely to be rare compared to four-state nucleotide sequence data [20]. Second, it is becoming increasingly apparent that the application of mitochondrial sequences and gene order data to questions of evolutionary history and phylogenetic relatedness requires a better understanding of the evolutionary dynamics of mt genomes [21]. Basic mechanisms of gene rearrangement associated with slipped-strand mispairing [22], errors in replication origins or end points [23], and intramolecular recombination [24], remain poorly understood. Likewise, tRNA remolding and tRNA recruitment events [25-28], gene rearrangement "hotspots" [29,30], the non-random loss of duplicated genes [31] and gene order homoplasy [32-34], which can act to confound phylogenetic inferences based on mtDNA sequences and gene orders, need to be explored more fully. Comparison of gene arrangements at low taxonomic levels can help to elucidate the process of gene rearrangement. For instance, the signature of specific processes such as tRNA remolding or recruitment can be most easily recognized when such events have occurred recently, since remolded or recruited tRNAs can be identified through high similarity scores and phylogenetic analyses [27,28]. Likewise, those taxonomic groupings with unusually labile genomes offer the opportunity to investigate the mechanics of gene rearrangement: telltale vestiges of gene duplication and translocation, typically erased or overwritten with time, may still be present within these genomes [35] and such intermediate stages can be critical to reconstructing the processes through which such gene rearrangements have occurred. Comparisons of mt genomes at low taxonomic levels, even within families and genera, can thus be extremely helpful in interpreting the evolutionary dynamics of these genomes and exploiting the phylogenetic signal retained within these DNA molecules [16].

Here we present further evidence of highly dynamic molluscan mt genomes by revealing extensive gene order changes within members of one caenogastropod family: the Vermetidae. Vermetids are a group of sessile, irregularly coiled, suspension-feeding gastropods found in warm temperate to tropical oceans around the world that radiated from a basal caenogastropod stock in the early Cenozoic Era. They are currently classified as members of the Hypsogastropoda [36,37], a large and diverse group with a fossil record extending back to the Permo-Triassic boundary that includes all extant caenogastropods, except for the Architaenioglossa, Cerithioidea and Campaniloidea. While relationships within the Hypsogastropoda are not well resolved, vermetids are typically positioned within the infraorder Littorinimorpha. More specifically, molecular analyses suggest that vermetids are members of a largely asiphonate clade of gastropods including the Littorinidae, Eatoniellidae, Rissoidae, Anabathridae, Hipponicidae, Pterotracheidae, Epitoniidae, Cerithiopsidae, Eulimidae, and Naticidae [37]. This association is also supported by morphological similarities in euspermatozoa shared by many members of this clade [38].

Gene order rearrangements have been recognized previously in this family [18] based on small (<3.5 kb) portions of the mt genome sequenced from several species within the genus *Dendropoma*. In this paper, we expand upon these earlier results by providing complete mt genomes for two *Dendropoma *species as well as for representatives of two other vermetid genera, *Thylacodes *and *Eualetes*. We also reveal additional gene rearrangements within this family through the partial genomes of the vermetid genera *Thylaeodus *and *Vermetus*. The extent of gene rearrangement within the family offers great potential for improving our phylogenetic hypothesis for the enigmatic Vermetidae as well as for understanding more fully the mechanics of gene order change within metazoan mt genomes.

## Methods

### Amplification and sequencing

Selection of taxa was based on: 1) the discovery of novel gene orders in these species following PCR amplifications spanning gene boundaries [18] and 2) those genomes that successfully amplified using long and accurate PCR (LAPCR). The collecting locality, tissue source, and Field Museum of Natural History (FMNH) voucher information for each specimen, are presented in Table 1, along with GenBank accession numbers, primer sequences, and lengths of amplification products. Other gene arrangements have been identified in additional vermetids based on partial genome sequences (<3 kb), but these results are not presented here (Rawlings *et al*., in prep).

**Table 1:** Sampling localities, tissue sources, and PCR primer sequences for the six vermetid species examined in this study

Species	FMNH voucher # GenBank accession #	Locality	Tissue	Primer Set A (5'-3') rrnL-F/cox1-R	Fragment Size (bp)	Primer Set B (5'-3') rrnL-R/cox1-F	Fragment Size (bp)
*Dendropoma maximum *(Sowerby1825)	FMNH 318221 HM174253	Gulf of Aqaba, Jordan	Foot tissue; buccal mass	CGAATTGAAAGGGGGGCTTGTGACCTCGATGTTG TTTCGATCCGTTAAAAGCATAGTGATAGCTCC	6877	ACGCTACCTTCGCACGGTCAAAGTACCGCGGC TTGTTATGCCAATAATGATTGGTGGTTTCGG	8809
*Dendropoma gregarium *Hadfield & Kay 1972	FMNH 318222 HM174252	HI, USA	Foot tissue; buccal mass	CAAATCGAAAAAAGGGTTTGCGACCTCGATGTTG TTACGGTCAGTTAAGAGTATGGTAATAGCACC	6490	ATGCTACCTTTGCACGGTCAGGGTACCGCGGC TGGTAATACCCATGATAATTGGAGGTTTTGG	9254
*Eualetes tulipa *(Chenu1843)	FMNH 318223 HM174254	Peanut Island, Palm Beach Co., FL, USA	Buccal mass	CATATCGAAAGAATAGTTTGCGACCTCGATGTTG TTTCGGTCCGTCAACAATATTGTAATTGCCCC	6880	TTCAACGAGAGCGACGGGCGATATGTACAC (rrnS-R) TGGTAATGCCTATAATGATTGGGGGGTTCGG	7472
*Thylacodes squamigerus*^1 ^(Carpenter, 1857)	FMNH 318997 HM174255	Corona del Mar, CA, USA	Foot tissue	CCCATCGAAAGAAGAGTTTGTGACCTCGATGTTG TTTCGGTCCGTCAACAGCATAGTAATAGCTCC	6648	ATGCTACCTTTGCACGGTCAGAGTACCGCGGC TGGTTATACCAATAATAATTGGTGGCTTCGG	9034
*Thylaeodus* *sp*.	FMNH 318224 HM174256	Kewalo Marine Laboratory, HI, USA	Head, foot & mantle margin	CATATTGAAAAAAAAGTTTGTGACCTCGATGTTG TTTCGATCAGTCAATAACATAGTAATTGCGCC	4703	ATGCTACCTTTGCACGGTCAGAGTACCGCGGC TAGTAATACCTATAATAATTGGTGGATTTGG	N/A
*Vermetus erectus *Dall, 1888	FMNH 318225 HM174257	Pourtales Terrace, Florida Keys; FL, USA	Foot tissue; buccal mass	CTAATCGAAGAAAAGGCTTGTGACCTCGATGTTG TTTCGGTCAGTCAGTAGTATAGTAATAGCACC	3107	ATGCTACCTTAGCACGGTTAAAATACCGCGGC TTGTCATGCCTATAATAATTGGCGGATTTGG	N/A

^1^*Thylacodes *has been demonstrated to have priority over *Serpulorbis*; hence *Serpulorbis squamigerus *is now correctly referred to as *Thylacodes squamigerus *(see [62]).

DNA was extracted from ethanol-preserved tissues using a phenol chloroform extraction protocol as described in [39]. Initially, an 1800 bp region of the mitochondrial genome spanning the *rrnS *to *nad1 *region was amplified as part of a phylogenetic analysis of Vermetidae [18]. This sequence was subsequently used to design outwardly facing primers for LAPCR (Table 1: rrnL-F; rrnL-R). Because attempts to amplify the genome in one piece were not successful, we amplified a 650 bp fragment of *cox1 *using modifications of Folmer's widely used *cox1 *primers [40]. This *cox1 *sequence was then used as a template for designing a second pair of primers (cox1-F; cox1-R). Successful amplifications were associated with the primer combinations: rrnL-F/cox1-R ("A" fragment) and rrnL-R/cox1-F ("B" fragment). LAPCR reactions were undertaken using GeneAmp XL PCR kits (Applied Biosystems; N8080193). Typically, 25 μL reactions were set up in two parts separated by a wax bead following the manufacturer's recommendations, using a [Mg(OAc)_2_] of 1.2 mM and an annealing temperature specific to the primer combination. Typical conditions consisted of a 94°C denaturation period lasting 60 s, followed by: 16 cycles at 94°C for 25 s, 60°C for 60 s, and 68°C for 10 min; 18 cycles at 94°C for 25 s, 60°C for 60 s, and 68°C for 12 min; and a final extension period at 72°C for 10 min. Amplifications were run on a Stratagene Gradient Robocycler. PCR products were cleaned by separating high molecular weight products from primers and sequencing reagents using Millipore Ultrafree filter columns [7]. Samples were added to 200 μL of sterile water and then spun in a picofuge for 15 min or until the filter membranes were dry. PCR products were eluted from the membrane in 20 μL of sterile distilled water, and 5 μL of this product was run out on a 0.8% agarose gel to confirm the presence of a band of the appropriate size. Typically, products from several replicate PCR reactions were pooled prior to quantitation. Once 3 ng of PCR product had been obtained, samples were dried down in a vacuum centrifuge and sent to the Joint Genome Institute, Walnut Creek, CA, where they were sequenced using standard shotgun sequencing protocols [7,8].

### Genome annotation and analysis

#### Genome annotation

The approximate locations of the rRNA and protein-encoding genes were determined by aligning each unannotated genome with genes from other caenogastropod mt genomes. The precise boundaries of rRNA genes could not be determined due to the lack of sequence similarity at the 5' and 3' ends; therefore, the location of each rRNA gene was assumed to extend from the boundary of the upstream flanking gene to the boundary of the downstream flanking gene, as in [41]. A standard initiation codon was located at the beginning of each protein-encoding gene (either ATG, ATA, or GTG), and the derived amino acid sequence was aligned with homologous protein sequences to ensure that this was a suitable initiation codon (based on length). When possible, the first proper stop codon (TAG or TAA) downstream of the initiation codon was chosen to terminate translation; however, to reduce overlap with downstream genes, abbreviated stop codons (T or TA) were selected for some genes. In these instances, polyadenylation of the mRNA was assumed to restore a full TAA stop codon [7]. Regardless, some genes appeared to overlap based on the conservation of their open reading frame sequences and the lack of a potential abbreviated stop codon. Gene locations and secondary structures of tRNAs were identified using tRNAscanSE [42] and ARWEN [43]. On the rare occasion that these programs did not find all of the expected tRNAs, the remaining tRNAs were found by eye and folded manually. We produced tRNA drawings manually using Canvas (ACD Systems). Using *cox1 *as the conventional starting point for the four genomes, linear maps of the circular genomes were created to facilitate comparison of gene orders amongst vermetids, across available caenogastropods, and between caenogastropods and other select molluscs, including the abalone, *Haliotis rubra *(Class Gastropoda,Superorder Vetigastropoda), the octopus, *Octopus vulgaris *(Class Cephalopoda), and the chiton, *Katharina tunicata *(Class Polyplacophora).

#### Nucleotide composition

The nucleotide composition of each complete and partial genome was described by calculating the overall base composition, %AT content, AT-skew, and GC-skew for the strand encoding *cox1*, hereafter referred to as the "+" strand. Base composition and %AT content were determined using MacVector (MacVector, Inc.), and strand skews (AT skew = (A-T)/(A+T); GC skew = (G-C)/G+C)) were calculated using the formulae of [44]. For complete genomes, the %AT content, AT-skew, and GC-skew were also calculated for rRNA genes, protein-encoding genes (for all bases, third codon positions, and third positions of four-fold degenerate (4FD) codons, as in [44]), tRNA genes (separately for those coded for on the "+" and "-" strand), and intergenic (unassigned) regions. Values were compared across categories within each genome, and within categories across genomes. In addition, we explored the nucleotide composition at third positions of 4FD codons in relation to the position of each protein-encoding gene within the genome [45]. Gene positions were determined by calculating the distance (number of nucleotides) from the midpoint of each gene to a reference point chosen here as the start of *nad1*.

A base composition plot (%A+C and %G+T along the length of the genome) was created for each complete genome using a sliding window of 100 nucleotides. Typically, the leading strand is G+T rich associated with its protracted single-stranded state during replication and transcription; deviations from this pattern can signify switches in the assignments of these strands [41]. Plots were aligned with linear representations of the matching genome to signify base composition trends for protein and tRNA genes encoded on the "+" strand, tRNA genes encoded on the "-" strand, and rRNA genes.

#### Genetic code

To ensure that all codons were being utilized, codon usage frequencies were analyzed using MacVector (MacVector, Inc.); unused codons can potentially signal a change in the genetic code [46]. Codons whose amino acid identity has changed in the mt genetic code of other metazoans (e.g. flatworms, echinoderms, and hemichordates; [47]) were investigated using a procedure similar to [48]: the derived amino acids of five codons (TGA, ATA, AGA, AGG, AAA) were examined in the alignment of three highly conserved proteins (*cox1*, *cox2*, and *cox3*) from the mt genomes of four vermetids, *D. gregarium*, *D. maximum*, *E. tulipa *and *T. squamigerus*, and several other caenogastropods. Within the vermetids, if the derived amino acid from any of these five codons occurred in a conserved position (present at that location in >50% of caenogastropod sequences examined) this was scored as a positive result. Amino acids that occurred in a non-conserved position (<50%) were scored as negative. The proportion of appearances in conserved *vs*. non-conserved locations was calculated for each codon across the vermetids examined. High percentages in conserved locations likely indicate the retention of amino acid identity by the specific codon [48]; low percentages can be suggestive of a change in amino acid identity, although other explanations are also possible.

#### Unassigned regions

As metazoan mt genomes are typically compact with minimal non-coding DNA [49], unassigned stretches of nucleotides often contain control elements for transcription or replication or remnants of duplicated protein, rRNA or tRNA genes. We examined putative non-coding regions (> 20 bp) for gene remnants, repeat sequences, inverted repeats, palindromes, and secondary structure features, all of which can be associated with signaling elements of the mt genome. Protein, rRNA and tRNA remnants were identified by aligning unassigned regions with annotated genes from caenogastropod mitochondrial genomes. MEME [50] and M-Fold [51] were used to identify potential sequence motifs and structural features, respectively.

#### tRNA remolding/recruitment

To search for close matches between tRNA genes indicative of gene remolding/recruitment, each tRNA gene sequence was aligned to tRNA genes from other available caenogastropod genomes (including new vermetid tRNA sequences). Similarity scores were based on initial tRNA alignments undertaken in MacVector that were subsequently adjusted by eye according to secondary structure features (stems vs. loops). As in [28], the third base of the anticodon triplet was excluded in the calculation of % similarity between two tRNA genes, but gaps were counted as mismatches.

## Results and Discussion

### Caenogastropod mitochondrial genomes

To date, comparisons across published caenogastropod mt genomes have suggested a model of gene order conservation unusual for the Gastropoda [15,48,52,53]. Among the 16 complete caenogastropod genomes available at NCBI as of January 8, 2010, gene order rearrangements within this clade appear minor, involving only changes in position of *trnL_UUR_*, *trnL_CUN_*, *trnV *and *trnS2*, and an inversion of *trnT *([53]). Likewise, only two rearrangements, one inversion and one transposition, separate the reconstructed ancestral gastropod gene order from that of most caenogastropods [15,53]. While this conservation in gene order within the Caenogastropoda may be real, it could also reflect a strong sampling bias for members of the Neogastropoda (12 complete genomes) - a largely Cenozoic radiation of predatory snails - with only four complete genomes from two genera (*Cymatium *and *Oncomelania*), examined from other caenogastropod groups. In contrast, wider sampling of mt gene orders within the Heterobranchia, a sister clade to the Caenogastropoda, has demonstrated highly dynamic mt genomes [15]. Of 13 genomes sampled across a disparate array of taxa, including 5 opisthobranchs (including an unpublished *Elysia *genome), 7 pulmonates (including a second *Biomphalaria *genome not included in [15]), and 1 basal heterobranch [15], numerous changes in gene order have been observed, with few mt gene boundaries shared between heterobranchs and the hypothetical ancestral gastropod mt genome inferred by [15].

**Figure 1:**
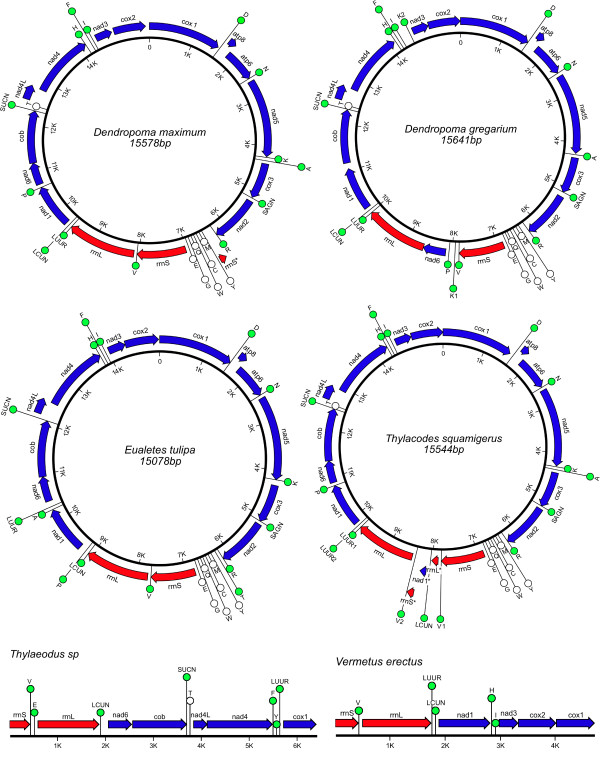
**Gene maps of four complete and two partial vermetid mt genomes**. Blue arrows represent protein-encoding genes; red arrows represent rRNA genes; and circles represent tRNA genes. Arrows indicate the direction of transcription (clockwise = "+" strand). Genes are labeled according to their standard abbreviations. For tRNA genes, green circles represent those genes located on the "+" strand and white circles those located on the "-" strand. Regions of unassigned nucleotides (presumed non-coding sequences) are not identified on these maps. As is the standard convention for metazoan mt genomes, *cox1 *has been designated the start point for the "+" strand. The asterisks (*) in *D. maximum *and *T. squamigerus *denote stretches of sequences (presumed pseudogenes) with high similarity to portions of complete genes. For exact locations of each gene, as well as unassigned regions, see Tables 2-7.

Here, based on a detailed sampling of mt genomes within one family of caenogastropods outside the Neogastropoda, we provide a new and very different picture of gene order dynamics within the Caenogastropoda. Our results increase the number of caenogastropod mt genomes sequenced to 20, substantially increase the sampling of mt genomes outside the Neogastropoda, and present the first direct evidence of major gene order rearrangements within the Littorinimorpha based on complete genome sequences. Full mt genomes were successfully sequenced for the vermetids, *Dendropoma gregarium*, *D. maximum*, *Eualetes tulipa*, and *Thylacodes squamigerus *(Figure 1; Tables 2, 3, 4 and 5). Amplifications of the "B" fragment (cox1-F/rrnL-R) were not successful for *Thylaeodus sp*. and *V. erectus *and consequently only partial mt genomes are described for these two taxa (Figure 1; Tables 6 and 7). Nevertheless, extensive gene rearrangements were evident within these species compared to other vermetids and caenogastropods. Characterization of the vermetid mt genomes presented below is based only on the four complete genomes, except where noted otherwise.

**Table 2:** Detailed description of genes and unassigned regions (UR) within the complete mt genome (15578 bp) of *Dendropoma maximum*

Gene	Start^a^	End	Length	Amino acids	Start codon	End codon^b^	UR (%AT)^c^
*cox1*	1	1536	1536	512	ATG	TAG	38 (76.3)
*trnD*	1575	1642	68	-	-	-	0
*atp8*	1643	1798	156	52	ATG	TAG	-7
*atp6*	1792	2479	688	229	ATG	T--	0
*trnN*	2480	2550	71	-	-	-	0
*nad5*	2551	4251	1701	567	ATG	TAA	14 (78.6)
*trnK*	4266	4340	75	-	-	-	-2
*trnA*	4339	4404	66	-	-	-	1
*cox3*	4406	5185	780	260	ATG	TAA	15 (86.7)
*trnS_AGN_*	5201	5274	74	-	-	-	1
*nad2*	5276	6295	1020	340	ATG	TAG	0
*trnR*	6296	6364	69	-	-	-	11 (54.5)/**167 (69.3)^d^**
rrnS* (pseudo)	6376	6496	121	-	-	-	35 (74.3)
trnY*	6532	6598	67	-	-	-	0
trnM*	6599	6668	70	-	-	-	2
trnC*	6671	6739	69	-	-	-	0
trnW*	6740	6807	68	-	-	-	-4
trnQ*	6804	6874	71	-	-	-	0
trnG*	6875	6943	69	-	-	-	0
trnE*	6944	7016	73	-	-	-	0
*rrnS*	7017	8049	1033	-	-	-	0
*trnV*	8050	8116	67	-	-	-	0
*rrnL*	8117	9563	1447	-	-	-	0
*trnL_UUR_*	9564	9633	70	-	-	-	-4
*trnL_CUN_*	9630	9698	69	-	-	-	1
*nad1*	9700	10632	933	311	ATG	TAA	2
*trnP*	10635	10707	73	-	-	-	1
*nad6*	10709	11189	481	160	ATG	T--	0
*cob*	11190	12329	1140	380	ATG	TAG	2
*trnS_UCN_*	12332	12399	68	-	-	-	33 (54.5)
trnT*	12433	12508	76	-	-	-	8
*nad4L*	12517	12810	294	98	ATG	TAG	-7
*nad4*	12804	14161	1358	452	GTG	TA-	0
*trnH*	14162	14231	70	-	-	-	46 (47.8)
*trnF*	14278	14346	69	-	-	-	0
*trnI*	14347	14418	72	-	-	-	2
*nad3*	14421	14774	354	118	ATG	TAG	46 (65.2)
*cox2*	14821	15498	678	226	ATG	TAG	80 (63.8)

^a ^Genes are arranged relative to *cox1*. Those encoded on the "-" strand are indicated with an asterisk (*).

^b ^T, and TA, refer to instances where incomplete stop codons are inferred.

^c ^Unassigned regions are identified by positive values. Negative values indicate overlap between adjacent genes. Values in brackets refer to %AT of the associated UR (only those UR >10 bp in length were analyzed).

^d ^For the unassigned region between *trnR *and *trnY *spanning the pseudogene *rrnS*.

**Table 3:** Detailed description of genes and unassigned regions (UR) within the complete mt genome (15641 bp) of *Dendropoma gregarium*

Gene	Start^a^	End	Length	Amino acids	Start codon	End codon^b^	UR (%AT)^c^
*cox1*	1	1533	1533	511	ATG	TAG	3
*trnD*	1537	1611	75	-	-	-	2
*atp8*	1614	1769	156	52	ATG	TAA	-7
*atp6*	1763	2452	690	230	ATG	TAA	7
*trnN*	2460	2535	76	-	-	-	2
*nad5*	2538	4259	1722	574	ATA	TAA	27 (74.1)
*trnA*	4287	4354	68	-	-	-	1
*cox3*	4356	5134	779	260	ATG	TA-	0
*trnS_AGN_*	5135	5201	67	-	-	-	38 (60.5)
*nad2*	5240	6252	1013	338	ATG	TA-	0
*trnR*	6253	6321	69	-	-	-	55 (72.7)
trnY*	6377	6443	67	-	-	-	1
trnM*	6445	6514	70	-	-	-	10 (30.0)
trnC*	6525	6597	73	-	-	-	1
trnW*	6599	6667	69	-	-	-	11 (50.0)
trnQ*	6679	6735	57	-	-	-	-3
trnG*	6733	6798	66	-	-	-	2
trnE*	6801	6872	72	-	-	-	0
*rrnS*	6873	7851	979	-	-	-	0
*trnV*	7852	7922	71	-	-	-	16 (50.0)
*trnK_1_*	7939	8013	75	-	-	-	55 (65.5)
*trnP*	8069	8139	71	-	-	-	1
*nad6*	8141	8629	489	163	ATG	TAG	0
*rrnL*	8630	9978	1349	-	-	-	0
*trnL_UUR_*	9979	10049	71	-	-	-	-2
*trnL_CUN_*	10048	10116	69	-	-	-	2
*nad1*	10119	11049	931	310	ATG	T--	125 (52.8)
*cob*	11175	12323	1149	383	ATG	TAA	13 (84.6)
*trnS_UCN_*	12337	12407	71	-	-	-	4
tRNA T*	12412	12479	68	-	-	-	7
*nad4L*	12487	12780	294	98	ATG	TAA	-7
*nad4*	12774	14129	1356	452	ATG	TAG	30 (66.7)
*trnH*	14160	14227	68	-	-	-	0
*trnF*	14228	14296	69	-	-	-	6
*trnI*	14303	14375	73	-	-	-	137 (58.4)
*trnK_2_*	14513	14586	74	-	-	-	2
*nad3*	14589	14939	351	117	ATG	TAG	7
*cox2*	14947	15636	690	230	ATG	TAG	5

^a ^Genes are arranged relative to *cox1*. Those encoded on the "-" strand are indicated with an asterisk (*).

^b ^T, and TA, refer to instances where incomplete stop codons are inferred.

^c ^Unassigned regions are identified by positive values. Negative values indicate overlap between adjacent genes. Values in brackets refer to %AT of the associated UR (only those UR >10 bp in length were analyzed).

**Table 4:** Detailed description of genes and unassigned regions (UR) within the complete mt genome (15078 bp) of *Eualetes tulipa*

Gene	Start^a^	End	Length	Amino acids	Start codon	End codon^b^	UR (%AT)^c^
*cox1*	1	1531	1531	510	ATG	T--	0
*trnD*	1532	1596	65	-	-	-	0
*atp8*	1597	1752	156	49	ATG	TAG	-7
*atp6*	1746	2430	685	228	ATG	T--	0
*trnN*	2431	2497	67	-	-	-	0
*nad5*	2498	4205	1708	569	ATG	T--	0
*trnK*	4206	4270	65	-	-	-	51 (62.1)
*cox3*	4322	5104	783	261	ATG	TAA	0
*trnS_AGN_*	5105	5168	64	-	-	-	0
*nad2*	5169	6176	1008	336	ATG	TAA	0
*trnR*	6177	6242	66	-	-	-	4
*trnT*	6247	6312	66	-	-	-	33 (66.7)
trnY*	6346	6411	66	-	-	-	2
trnM*	6414	6480	67	-	-	-	0
trnC*	6481	6546	66	-	-	-	0
trnW*	6547	6609	63	-	-	-	-5
trnQ*	6605	6671	67	-	-	-	0
trnG*	6672	6735	64	-	-	-	6
trnE*	6742	6807	66	-	-	-	0
*rrnS*	6808	7716	909	-	-	-	0
*trnV*	7717	7783	67	-	-	-	0
*rrnL*	7784	9087	1304	-	-	-	0
*trnL_CUN_*	9088	9152	65	-	-	-	9
*trnP*	9162	9225	64	-	-	-	73 (71.2)
*nad1*	9299	10231	933	311	ATA	TAG	10 (50.0)
*trnA*	10242	10304	63	-	-	-	2
*trnL_UUR_*	10307	10371	65	-	-	-	56 (58.9)
*nad6*	10428	10934	507	169	ATG	TAG	2
*cob*	10937	12076	1140	380	ATG	TAG	0
*trnS_UCN_*	12077	12143	67	-	-	-	18 (66.7)
*nad4L*	12162	12452	291	97	ATG	TAG	-7
*nad4*	12446	13810	1365	455	ATG	TAG	-1
*trnH*	13810	13875	66	-	-	-	-1
*trnF*	13875	13940	66	-	-	-	0
*trnI*	13941	14012	72	-	-	-	0
*nad3*	14013	14366	354	118	ATG	TAG	3
*cox2*	14370	15058	689	229	ATG	TA-	20 (65.0)

^a ^Genes are arranged relative to *cox1*. Those encoded on the "-" strand are indicated with an asterisk (*).

^b ^T, and TA, refer to instances where incomplete stop codons are inferred.

^c ^Unassigned regions are identified by positive values. Negative values indicate overlap between adjacent genes. Values in brackets refer to %AT of the associated UR (only those UR >10 bp in length were analyzed).

**Table 5:** Detailed description of genes and unassigned regions (UR) within the complete mt genome (15544 bp) of *Thylacodes squamigerus*

Gene	Start^a^	End	Length	Amino acids	Start codon	End codon^b^	UR (%AT)^c^
*cox1*	1	1537	1537	512	ATG	T--	78 (57.5)
*trnD*	1616	1682	67	-	-	-	1
*atp8*	1684	1839	156	52	ATG	TAG	-7
*atp6*	1833	2531	699	233	ATG	TAA	12 (83.3)
*trnN*	2544	2610	67	-	-	-	1
*nad5*	2612	4306	1695	565	ATG	TAG	17 (47.1)
*trnK*	4324	4389	66	-	-	-	-3
*trnA*	4387	4453	67	-	-	-	0
*cox3*	4454	5236	783	261	ATG	TAA	-2
*trnS_AGN_*	5235	5299	65				0
*nad2*	5300	6298	999	333	ATG	TAG	-1
*trnR*	6298	6365	68	-	-	-	35 (74.3)
trnY*	6401	6467	67	-	-	-	-2
trnM*	6466	6531	66	-	-	-	6
trnC*	6538	6599	62	-	-	-	2
trnW*	6602	6669	68	-	-	-	-5
trnQ*	6665	6729	65	-	-	-	8
trnG*	6738	6802	65	-	-	-	3
trnE*	6806	6873	68	-	-	-	0
*rrnS*	6874	7809	936	-	-	-	0
*trnV_1_*	7810	7877	68	-	-	-	0/**163 (69.9)^d^**
*rrnL *(pseudo)	7878	8017	140	-	-	-	23 (82.6)
*trnL_CUN_*	8041	8105	65	-	-	-	0/**270 (58.9)^e^**
*nad1 *(pseudo)	8106	8225	120	-	-	-	36 (61.1)
*rrnS *(pseudo)	8262	8375	114	-	-	-	0
*trnV_2_*	8376	8443	68	-	-	-	0
*rrnL*	8444	9733	1290	-	-	-	0
*trnL_UUR1_*	9734	9801	68	-	-	-	-4
*trnL_UUR2_*	9798	9862	65	-	-	-	0
*nad1*	9863	10798	936	312	ATG	TAG	1
*trnP*	10800	10867	68	-	-	-	2
*nad6*	10870	11350	481	160	ATG	T--	0
*cob*	11351	12490	1140	380	ATG	TAG	0
*trnS_UCN_*	12491	12561	71	-	-	-	-2
trnT*	12560	12623	64	-	-	-	5
*nad4L*	12629	12919	291	97	ATG	TAG	-7
*nad4*	12913	14277	1365	455	ATG	TAA	5
*trnH*	14283	14344	62	-	-	-	2
*trnF*	14347	14416	70	-	-	-	6
*trnI*	14423	14492	70	-	-	-	2
*nad3*	14495	14848	354	118	ATG	TAG	6
*cox2*	14855	15544	690	230	ATG	TAA	0

^a ^Genes are arranged relative to *cox1*. Those encoded on the "-" strand are indicated with an asterisk (*).

^b ^T, and TA, refer to instances where incomplete stop codons are inferred.

^c ^Unassigned regions are identified by positive values. Negative values indicate overlap between adjacent genes. Values in brackets refer to %AT of the associated UR (only those UR >10 bp in length were analyzed).

^d ^For the unassigned region between *trnV_1 _*and *trnL_CUN _*spanning the pseudogene *rrnL*.

^e ^For the unassigned region between *trnL_CUN _*and *trnV_2 _*spanning the pseudogenes *nad1 *and *rrnS*.

**Table 6:** Detailed description of genes and unassigned regions (UR) within the partial mt genome of *Thylaeodus sp.*

Gene	Start^a^	End	Length	Amino acids	Start codon	End codon^b^	UR (%AT)^c^
*rrnS *(partial)	1	431	431	-			5
*trnV*	437	504	68	-			13 (53.8)
*trnE*	518	591	74	-			0
*rrnL*	592	1882	1291	-			16 (56.3)
*trnL_CUN_*	1899	1964	66	-			99 (70.7)
*nad6*	2064	2562	499	166	ATG	T--	0
*cob*	2563	3703	1141	380	ATG	T--	-6
*trnS_UCN_*	3698	3766	69	-			1
trnT*	3768	3834	67	-			11 (100.0)
*nad4L*	3846	4131	286	95	ATG	T--	-2
*nad4*	4130	5491	1362	454	ATG	TAA	6
*trnF*	5498	5565	68	-	-	-	9
*trnY*	5575	5639	65	-	-	-	-1
*trnL_UUR_*	5639	5708	70	-	-	-	11 (63.6)
*cox1 *(partial)	5720	6415	696	232	ATG	N/A	0

^a ^Genes are not arranged relative to *cox1 *because only a partial genome is available. Those genes encoded on the "-" strand are indicated with an asterisk (*).

^b ^T, and TA, refer to instances where incomplete stop codons were inferred.

^c ^Unassigned regions are identified by positive values. Negative values indicate overlap between adjacent genes. Values in brackets refer to %AT of the associated UR (only those UR >10 bp in length were analyzed).

**Table 7:** Detailed description of genes and unassigned regions (UR) within the partial mt genome of *Vermetus erectus*

Gene	Start^a^	End	Length	Amino acids	Start codon	End codon^b^	UR (%AT)^c^
*rrnS*	1	429	429	-	-	-	0
*trnV*	430	499	70	-	-	-	0
*rrnL*	500	1756	1257	-	-	-	0
*trnL_UUR_*	1757	1819	63	-	-	-	3
*trnL_CUN_*	1823	1887	65	-	-	-	1
*nad1*	1889	2824	936	312	ATG	TAG	18 (66.7)
*trnH*	2843	2904	62	-	-	-	10 (60.0)
*trnI*	2915	2980	66	-	-	-	0
*nad3*	2981	3331	351	117	ATG	TAG	3
*cox2*	3335	4022	688	229	ATG	T--	0
*cox1 *(partial)	4023	4718	696	232	ATG	N/A	0

^a ^Genes are not arranged relative to *cox1 *because only a partial genome is available. Those genes encoded on the "-" strand are indicated with an asterisk (*).

^b ^T, and TA, refer to instances where incomplete stop codons were inferred.

^c ^Unassigned regions are identified by positive values. Negative values indicate overlap between adjacent genes. Values in brackets refer to %AT of the associated UR (only those UR >10 bp in length were analyzed).

### Genome organization

The mitochondrial genomes of *D. gregarium*, *D. maximum*, *E. tulipa*, and *T. squamigerus *varied in size from 15078 bp to 15641 bp, similar in length to other caenogastropods (range: 15182 - 16648 bp, n = 16) but considerably larger than most heterobranchs (range: 13670 - 14745 bp, n = 13). Each genome contained the 37 genes typical of most animal mitochondrial genomes (Figure 1; Tables 2, 3, 4 and 5), with all 13 protein-encoding genes and 2 rRNA genes located on the "+" strand along with either 14 or 15 tRNA genes depending on the species. In each of the four genomes, the tRNA genes, *trnY, trnM, trnC, trnW, trnQ, trnG*, and *trnE*, were located on the "-" strand forming a cassette of 7 adjacent tRNAs. The only difference across genomes in the strand specific coding of a gene was for *trnT*, which was located on the "+" strand of *E. tulipa*, but on the "-" strand of *D. gregarium*, *D. maximum*, and *T. squamigerus*. The tendency for protein and rRNA genes to be coded for on the same strand has been found in all caenogastropod taxa examined so far, but this is atypical for molluscs (excluding bivalves) and other metazoan mt genomes described to date (137 of 1428 genomes as of Dec 17, 2008; [54]). The predominance of this single-strand-dependence for protein and rRNA genes among sponges and cnidarians has led to the proposition that this is the plesiomorphic metazoan condition [54].

The tRNA genes identified within each genome and their secondary structures are presented as additional files (see Additional file 1, Figure S1; Additional file 2, Figure S2; Additional file 3, Figure S3; Additional file 4, Figure S4; Additional file 5, Figure S5; Additional file 6, Figure S6). Extra tRNA genes were found in two vermetid genomes. *D. gregarium *had a second *trnK *located between genes *trnI *and *nad3 *(see Figure 1). This second *trnK *can be folded in to a typical tRNA cloverleaf structure (see Additional file 2, Figure S2), and thus may be functional. The genome of *T. squamigerus *also contained additional *trnV *and *trnL_UUR _*genes (see Figure 1). These genes were associated with two large stretches of unassigned sequence positioned between the two copies of *trnV *(see below).

Overlapping adjacent genes were common in vermetid mt genomes. Two pairs of protein-encoding genes overlapped in all four species (*atp8 *and *atp6*; *nad4L *and *nad4*); overlap of *nad4L *and *nad4 *was also evident in the partial genome of *Thylaeodus*. These gene pairs commonly overlap in animal mt genomes. This phenomenon was more variable between tRNA genes (see Tables 2, 3, 4, 5, 6 and 7 for specific examples). In addition, in three instances tRNA genes overlapped with protein-encoding genes (*trnH *and *nad4 *in *E. tulipa*; *trnS_AGN _*with *cox3 *and *trnR *with *nad2 *in *T. squamigerus*). Similar comparisons could not be made for the two rRNA genes since their boundaries were only imprecisely defined by boundaries with neighbouring genes.

#### Gene initiation and termination

Nearly all protein-encoding genes (58/61) were initiated by the canonical ATG start codon, although ATA (twice) and GTG (once) also acted as start codons (Tables 2, 3, 4, 5, 6 and 7). These start codons are not unusual in molluscan mt genomes, but their usage, as found elsewhere, appears to be much less frequent than ATG [7] (but see [15]). TAG was the most common termination codon (28/59), but TAA (15/59) and abbreviated stop codons (a single T or TA terminating the open reading frame; 16/59) were used slightly more frequently when considered together. The sequences for the *cox1 *genes of *Thylaeodus *and *Vermetus *were incomplete so the termination codons for these genes are currently unknown.

#### Unassigned regions

The number of nucleotides unassignable to any gene ranged from 289 (1.9% of the genome) in *Eualetes tulipa *to 625 (4.0% of the genome) in *Thylacodes squamigerus *(Table 8). No unassigned stretch of sequence was greater than 270 bp in length, although intergenic regions ranging in size from 10 - 99 bp were common (Table 8). There was little consistency in size and position of unassigned regions across genomes, with the position of the largest region found in different areas for each species. Short (≤ 15 bp) AT-rich (>80%) intergenic regions were evident within the genomes of *Dendropoma maximum*, *D. gregarium*, and *T. squamigerus*, but not in *E. tulipa *(Tables 2, 3, 4 and 5), and again, the position of these sequences varied across taxa. Stretches of unassigned sequence including inverted repetitive elements are known to reside between *trnF *and *cox3 *in the mt genomes of several caenogastropods, likely representing the control region for replication and transcription in these genomes [52,53]. In our comparisons across the four complete vermetid genomes, we were unable to identify any similar regions associated with repetitive sequence motifs or palindromes. As is common in mt genomes, however, secondary structure elements were found by MFOLD within many of these intergenic regions, suggestive of a role in post-transcriptional modification of the polycistronic transcript.

**Table 8:** A comparison of unassigned regions (UR) within the complete mt genomes of four vermetid gastropods

			UR length distribution^c^				Longest UR^c^	
**Species**	**% UR (bp)^a^**	**#^b^**	**10-19 bp**	**20-39 bp**	**40-99 bp**	**> 99 bp**	**bp**	**Location**

*Dendropoma maximum *^d^	2.95 (459)	8	2	2	3	1	167	*trnR - trnY*
*Dendropoma gregarium*	3.65 (571)	11	4	3	2	2	137	*trnI - trnK_2_*
*Eualetes tulipa*	1.92 (289)	7	2	2	3	0	73	*trnP - nad1*
*Thylacodes squamigerus *^d^	4.02 (625)	6	2	1	1	2	270	*trnL_CUN _- trnV_2_*

^a ^Expressed as a percent of the whole genome and the total number of base pairs.

^b ^Includes all unassigned sequences ≥ 10 bp in length.

^c ^The location and size of the UR is defined by upstream and downstream neighbouring genes.

^d ^Data for *Dendropoma maximum *and *Thylacodes squamigerus *include pseudogene sequences found within some unassigned regions (see Tables 2 and 5).

Our analyses did uncover several interesting features within large intergenic regions of *Dendropoma maximum *and *Thylacodes squamigerus *(Figure 1). In *D. maximum*, we identified a 121 bp stretch within an unassigned region of 167 bp between *trnR *and *trnY *that was a perfect match to the reverse complement of a portion of *rrnS*. Likewise, in *Thylacodes squamigerus*, in an unassigned region between *trnV_1 _*and *trnL_CUN_*, we found a 140 bp stretch of sequence that was identical to a corresponding portion of *rrnL*. In addition, between *trnL_CUN _*and *trnV_2_*, we discovered a 120 bp remnant of *nad1 *(82% identical, with a 91 bp stretch differing only in 6 bases) and a 114 bp remnant of *rrnS *(92% identical). These pseudogene fragments in *Thylacodes*, as well as the extra tRNAs (*trnV *and *trnL_UUR_*) present in this region, are likely the result of a duplication event spanning *rrnS-trnV-rrnL-trnL_UUR_-trnL_CUN_-nad1*, with subsequent overwriting of some gene duplicates (Rawlings *et al*., in prep).

#### Nucleotide composition and skews

**Figure 2:**
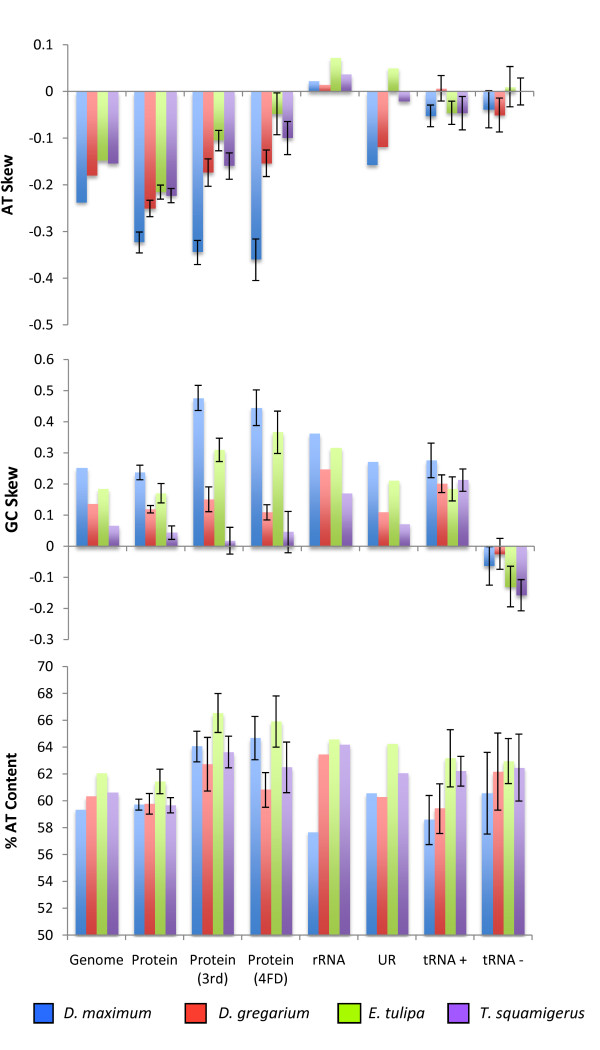
**Nucleotide composition of four complete vermetid genomes**. For each species, the %AT content, AT-skew, and GC-skew are shown for the "+" strand of the complete genome and its functional components, including: protein-encoding genes (complete protein, 3^rd ^codon positions only, and 3^rd ^positions of 4-fold degenerate (4FD) codons only), rRNA genes, unassigned regions (UR), and tRNA genes (including "+" and "-" strand encoded genes). Error bars refer to standard errors based on a sample size from each genome of: 13 protein encoding genes, 14 -15 "+" strand tRNA genes, and 7-8 "-" strand tRNA genes.

All four vermetid genomes were AT-rich, with these two nucleotides accounting for 59 - 62% of the genome (Figure 2; Additional file 7, Table S1); this trend was consistent across regions of each genome associated with protein-encoding genes, rRNA genes, tRNAs, and non-coding regions. Interestingly, however, the AT-biases of vermetid genomes were noticeably lower than those reported for other complete caenogastropod genomes (range: 65.2-70.1%, n = 14; Additional file 7, Table S1). Likewise, AT content was only moderately higher at third-codon positions and 4FD sites of protein-encoding genes, a result unusual in comparison with many other protostomes where %AT can often exceed >80% at 4FD sites [44]. Skew analyses revealed that the "+" strand was strongly biased against A (AT-skew ranging from -0.148 to -0.238) and towards G (GC-skew ranging from +0.065 to +0.251). This pattern was similar to other caenogastropods (Additional file 7, Table S1), heterobranchs [15], and the chiton, *Katharina tunicata *[55], but opposite to that found on the "+" strand of the abalone *Haliotis *[56] and the cephalopods, *Nautilus *and *Octopus*, suggestive of switches in the assignments of leading and lagging strands within the Mollusca [41].

Skew patterns were different between regions of the genome depending on their function. For AT-skews, the most strongly biased areas were found associated with protein-encoding genes, but these were not necessarily most pronounced at third positions or at 4FD sites (except for *D. maximum*; Figure 2). The two rRNA genes showed a marked difference from protein-encoding genes, with a slight positive AT-skew, on average. Differences in skew patterns between rRNA and protein-encoding genes have been noted elsewhere [41,57], and may reflect base-pairing constraints associated with the secondary structures of these rRNAs [41]. Unassigned regions, assumed to be non-coding, were expected to experience similar selective pressures to those of 4FD third codon positions; this correspondence was not strong, however, with unassigned regions showing variation in AT-bias across taxa from slight positive AT-skews to moderate negative skews. tRNA genes regardless of whether they were coded for on the "+" or "-" strand exhibited no consistent skew patterns, with an average close to zero.

GC-skews were moderately positive for most regions of the genome, including protein-encoding genes, rRNA genes, unassigned regions, and tRNAs encoded on the "+" strand. Skews were particularly strong, however, for the third codon positions and 4FD third codon positions in *Dendropoma maximum *and *Eualetes tulipa*. On average, neutral to negative GC-skews were associated with regions of the "+" strand associated with tRNA genes encoded on the "-" strand.

**Figure 3:**
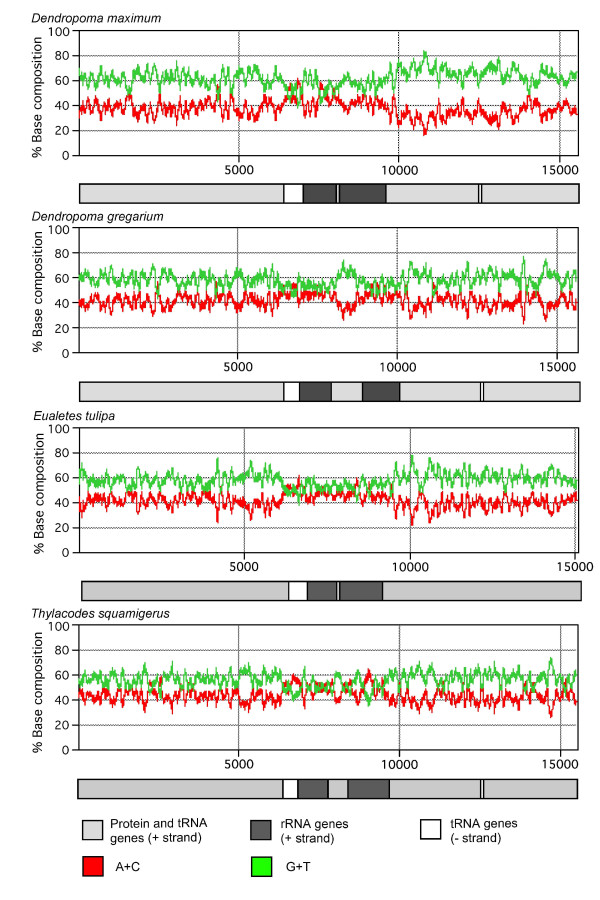
**Plots of A+C and G+T composition along the "+" strand of the mt genomes of *D. maximum, D. gregarium*, *E. tulipa*, and *T. squamigerus *using a sliding window of 100 nucleotides**. Each plot is positioned above a linear representation of its corresponding genome highlighting regions corresponding to the presence of: 1) protein encoding genes and tRNA genes encoded on the "+" strand, 2) rRNA genes encoded on the "+" strand, and 3) tRNA genes encoded on the "-" strand. See [41] for a comparison with *Nautilus *and *Katharina*. As is the standard convention for metazoan mt genomes, *cox1 *has been designated the start point for the "+" strand.

As predicted by skew patterns described above, G+T composition varied across the length of the "+" strand in all four complete genomes (Figure 3). Areas of weak or no G+T bias were typically associated with rRNAs and regions with tRNAs encoded on the "-" strand. *Dendropoma maximum *exhibited a particularly strong bias for G+T (>65%) in the region from 10,000 to 14,000 bp.

#### Codon frequency

No evidence was found of changes to the standard genetic code employed by other molluscs (Additional file 8, Table S2). Each codon was used multiple times in the protein-encoding genes of each species (Additional file 9, Table S3), however, codon usage frequencies were not equal. Codon usage strongly reflected skew patterns at the third position for synonymous codons (both two-fold and four-fold degenerate codons; see Additional file 9, Table S3): the most abundant base at the third codon position across all four complete genomes was T (36.7-43.1%) and the least abundant base was C (9.4-17.9%). The relative ranking of G (18.5-26.5%) and of A (21.0-29.8%), however, was less consistent across genomes.

#### Putative origins of replication

Locating the regions of the mt genomes associated with the replication origins (ORs, also known as control regions or A+T rich regions) for both "+" and "-" strands can be challenging based on a knowledge of the nucleotide sequence of mt genomes alone. Recognition elements can be in the form of conserved sequence blocks, regions rich in A+T, stable stem-loop structures containing T-rich loops, or repetitive elements/palindromes [58]. ORs can also be identified by association with rearrangement "hotspots" [23]. Such features are not definitive evidence, however, and can differ in utility across taxonomic groups. For instance, only 35% of tentatively identified molluscan control regions have been found to have palindromes and the average %AT content of these regions is in the range of 65-70%, close to the average genomic %AT content for molluscs (based on 9 molluscan genomes analyzed) [59]. Consequently, looking for palindromes or A+T rich regions may not be helpful in identifying the OR of molluscs. In contrast, 85% of insect control regions have palindromes and CR regions are associated with >80% AT [59].

Because none of the recognition elements described above were useful in locating putative ORs within the complete genomes of the four vermetids examined here, we attempted to locate ORs by examining the relationship between nucleotide composition and the position of protein-encoding genes within these genomes. Reyes et al. [45] determined that the AT and GC-skews at 4FD sites of protein-encoding genes of 25 mammalian mt genomes were significantly correlated with the duration of single stranded state of heavy-strand genes during replication, with increased duration reflecting, in part, the proximity of protein encoding genes to the origin of replication of the heavy strand (OR_H_), as well as their position relative to the OR of the light strand (OR_L_). Longer durations in the single stranded condition increase the vulnerability of DNAs to hydrolytic and oxidative damage creating the compositional asymmetries between the heavy and light strands (or leading and lagging strands). In vertebrates, the OR_L _is typically located two-thirds of the way around the genome from the OR_H_. In insects, the only protostomes examined in detail, however, the replication origins for both strands (leading and lagging) appear to be in close proximity, near the ends of a conserved block within the A+T rich region, with the lagging strand beginning after 95% of the leading strand has been replicated [60]. Comparative studies are lacking for molluscs.

**Figure 4:**
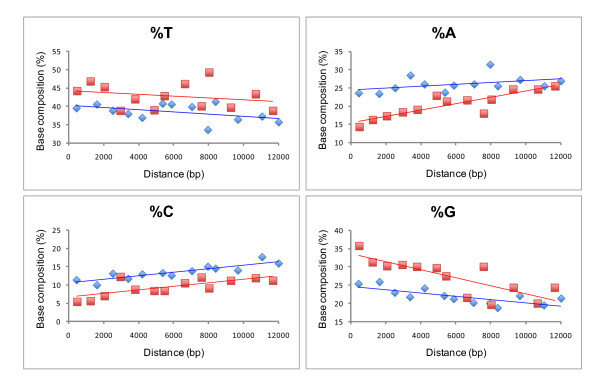
**Variation in nucleotide composition at 4FD sites of protein-coding genes in relation to the position of each gene within the genome**. Data are presented for: 1) the mean of all four vermetid genomes (diamonds) and 2) *Dendropoma maximum *alone (squares). The position of each protein encoding gene was calculated as the number of nucleotides from the beginning of *nad1 *(or *nad6 *for* D. gregarium*) to the midpoint of the target gene. This different start point for *D. gregarium *was based on the derived translocation of *nad6 *to a position directly upstream from *nad1 *in this taxon [18]. Regression equations are as follows. All four genomes: Y = -2.92 × 10^-4 ^X + 40.20, **r^2 ^= 0.21**[%T]; Y = 2.59 × 10^-4 ^X + 24.47, **r^2 ^= 0.18 **[%A]; Y = 4.84 × 10^-4 ^X + 10.60, **r^2 ^= 0.79 **[%C]; Y = -4.51 × 10^-4 ^X + 24.73, **r^2 ^= 0.56 **[%G]; *D. maximum *alone: Y = -2.45 × 10^-4 ^X + 44.22, **r^2 ^= 0.07 **[%T]; Y = 8.66 × 10^-4 ^X + 15.47, **r^2 ^= 0.78 **[%A]; Y = 4.83 × 10^-4 ^X + 6.63, **r^2 ^= 0.55 **[%C]; Y = -1.10 × 10^-3 ^X + 33.67, **r^2 ^= 0.65 **[%G].

If the ORs for both strands are located close to one another in molluscs, then examining nucleotide composition in relation to the position of each protein-encoding gene could reveal putative sites for the OR. We tested this by plotting nucleotide composition at 4FD third codon positions as in [45] versus the midpoint position of each protein-encoding gene (Figure 4). By moving the putative OR between different protein-encoding genes, we found a compelling linear relationship, with a negative slope for %T and %G and a positive slope for %A and %C, when we positioned the OR between *nad2 *and *nad1 *(or between *nad2 *and *nad6 *for *D. gregarium*). This region generally encompasses the cassette of 7 tRNA genes encoded on the "-" strand, two rRNA genes, as well as additional tRNAs, albeit with some notable differences amongst taxa (Figure 1). This relationship is shown based on averaged values across all four complete vermetid genomes (Figure 4: diamonds), and separately for the genome of *Dendropoma maximum *(Figure 4: squares) which exhibited the strongest pattern across all four taxa. Reyes et al. [45] found an increase in the frequency of A and C at 4FD sites in the light (sense) strand in direct relation to the single-stranded duration of the heavy strand. They speculated that this was the result of spontaneous deamination of adenine into hypoxanthine, which base pairs with C rather than T, and cytosine into uracil, which base pairs with A rather than G, along the heavy strand during its single-stranded state in replication (with associated increases in nucleotides A and C in the light, sense strand). In vermetid genomes, where genes are encoded on the opposite strand to vertebrates (i.e. the heavy strand is the sense or "+" strand for all vermetid protein-encoding genes, see Figure 3), genes with lower frequencies of G and T (or higher frequencies of C and A) at 4FD sites should be those experiencing shorter durations in the single-stranded condition. The marked difference in nucleotide composition of 4FD sites between *nad2 *(low %G and %T) and *nad1 *(high %G and %T) is thus suggestive of substantial differences in exposure of these two genes to the single-stranded condition. Consequently, the OR may lie between these two protein-encoding genes. Comparisons of nucleotide composition patterns along the genomes of protostome taxa with well defined ORs are now necessary to confirm or refute the predictive power of such analyses in identifying control regions.

Two other observations support the general location for the OR between *nad2 *and *nad1*. First, this region is associated with a major change in base compositional bias reflecting the presence of rRNA genes and a cassette of tRNAs encoded on the "-" strand (Figure 3). Such changes are thought to be associated with ORs [41,58]. Second, this region of the mt genome appears to be involved in a number of gene order rearrangements, possibly reflecting a rearrangement "hotspot" (see *Gene order rearrangements*, below). Hotspots have been associated with ORs in previous studies [23]. In many other caenogastropods, however, ORs have been tentatively identified in a different region of the genome, despite the presence of a similar cassette of tRNA genes located on the "-" strand [48,52,53]. In these taxa, the OR is thought to be present between *trnF *and *coxIII *within an unassigned stretch of sequence of variable length (from 15 - 848 bp) associated with inverted repeats and secondary structure elements. This gene boundary is not present in the Vermetidae. Direct examination of mRNAs is now needed to demonstrate conclusively the presence of ORs within vermetid and other caenogastropod mt genomes.

### Gene order rearrangements

**Figure 5:**
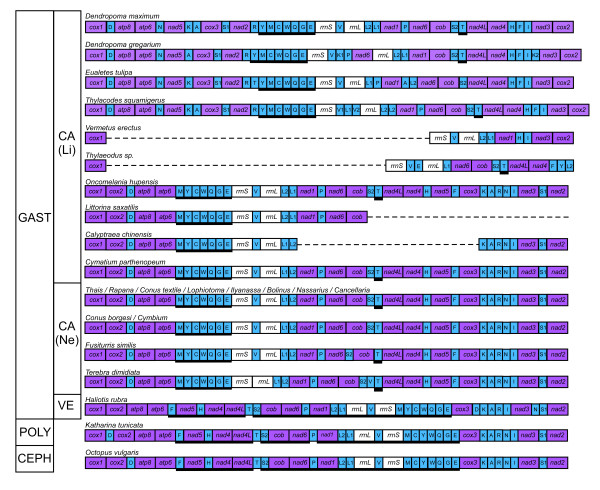
**Linear arrangement of protein-encoding, rRNA and tRNA genes within the complete mt genomes of vermetid gastropods, caenogastropods, and select representatives of other molluscan groups**. Partial genomes of *Thylaeodus sp, Vermetus erectus, Littorina saxatilis *and *Calyptraea chinensis *are also included. As is the standard convention for metazoan mt genomes, *cox1 *has been designated the start point for the "+" strand for all genomes. The dashed line indicates missing genes in the representation of partial genomes. Genes are transcribed left-to-right as depicted except for those underlined to signify opposite orientation. Gene names are as in Figure 1 except that *L1 *and *L2 *refer to *trnL_CUN _*and *trnL_UUR_*, respectively, and S1 and S2 refer to *trnS_AGN _*and *trnS_UCN_*, respectively. Pseudogenes identified in the genomes of *Dendropoma maximum *and *Thylacodes squamigerus *are not shown. In some instances, gene orders differ from their original publications (e.g., [53]); this is based on revised annotations listed in MitoZoa [63]. Abbreviations are as follows. GAST: Class Gastropoda; POLY: Class Polyplacophora; CEPH: Class Cephalopoda; CA: Caenogastropoda; Li: Littorinimorpha; Ne: Neogastropoda; VE: Vetigastropoda.

Each of the vermetid genomes examined here possessed a unique gene arrangement (Figure 5). The four complete vermetid genomes differed primarily in the position of tRNA genes, the most mobile elements within the mt genome [16]. While the position of many tRNA genes was conserved, the location of *trnA, trnK, trnP, trnT*, *trnL_CUN_*, *trnL_UUR_*, and *trnV *was more variable across the four genomes. Only *D. gregarium *differed in the order of protein-encoding genes, with *nad6 *changing position relative to other protein encoding and rRNA genes, as described in [18]. The two partial genomes of *Vermetus erectus *and *Thylaeodus sp*. provided additional evidence of the extent of gene rearrangement within this family, however, with novel arrangements both tRNA and protein-encoding genes.

Given the extent of shared gene boundaries with representatives of other molluscan classes, Grande et al. [15] have suggested that the mt genome of the abalone, *Haliotis rubra *[56], represents the ancestral gene order of gastropods, albeit with two derived changes. The position of *trnD *and *trnN *are likely autapomorphies of *Haliotis*, since many caenogastropods share a different (and presumed ancestral) position for *trnD *with *Octopus *and *trnN *with *Octopus *and *Katharina *(Figure 5). Based on this inferred plesiomorphic gastropod condition, vermetids exhibit a number of derived gene order changes, some of which are shared with other caenogastropods. All published caenogastropod mt genomes, and the four complete vermetid genomes examined here, share an inversion of a block of 23 genes spanning from *trnF *to *trnE *in *Haliotis*, with a reversion (to the original strand) of genes spanning *trnM *to *trnE *within this block. As discussed by [48], this rearrangement must have occurred before the divergence of caenogastropods, but after their separation from the Vetigastropoda (*Haliotis*).

Vermetid mt genomes also shared an interesting gene order change with two other members of the superfamily Littorinimorpha, *Littorina saxatilis *and *Oncomelania hupensis*, associated with the position of the two leucine tRNA genes, *trnL_UUR _*and *trnL_CUN _*(Figure 5). Within the mt genome of *Haliotis*, *Octopus*, and *Katharina*, these two leucine tRNAs are sandwiched between *rrnL *and *nad1 *in the following arrangement: *rrnL-trnL_CUN_-trnL_UUR_-nad1*. This gene order is retained in all caenogastropod mt genomes sampled to date, except for *Littorina, Oncomelania, Dendropoma maximum, D. gregarium*, and *Vermetus erectus*, where the relative position of these two genes has switched, such that *trnL_UUR _*is located directly upstream from *trnL_CUN_*. (Note: this gene order is incorrectly annotated in [48] (pg 40), and this gene order change is not recognized in [15](page 11) or [53](page 4), where it is stated that there are no differences in gene order between the partial genome of *Littorina *and complete genome of neogastropods). This difference in the order of these two tRNAs is also present in several other vermetid taxa (data not shown), but is not present in two additional members of the Littorinimorpha: *Cymatium parthenopeum *and *Calyptraea chinensis *[53]. While this shared gene rearrangement may be a synapomorphy defining a clade within the Littorinimorpha to which the Littorinidae (*Littorina*), Pomatiopsidae (*Oncomelania*) and Vermetidae belong, changes in tRNA positions involving neighbouring leucine tRNA genes should be treated with caution. Gene translocations among neighbouring genes appear to occur with increased frequency in mt genomes and thus may be more likely to arise independently [23]. Such "position switches" between neighbours may therefore be less reliable phylogenetic characters for addressing deeper level phylogenetic questions. In addition, simple changes in position of these two leucine tRNA genes can mask more complicated dynamics that may involve gene duplications and tRNA remolding events [28] (see *Evidence for tRNA remolding and recruitment*, below). Uncovering the dynamics that have occurred within this region of the genome can be essential to interpreting gene identity correctly and accurately reconstructing past gene rearrangement events. The discovery of two *trnL_UUR _*genes side by side between *rrnL *and *nad1 *of *Thylacodes squamigerus *further suggests that such tRNA remolding events may be at play within vermetid mt genomes.

Some gene order arrangements were unique to the Vermetidae (Figure 5). All four complete vermetid genomes shared a block of three protein-encoding genes and 3 - 4 tRNA genes: *trnN-nad5-trnK-trnA-cox3-trnS1-nad2*, with *trnA *and *trnK *absent from this block in *Eualetes *and *D. gregarium*, respectively. This gene rearrangement was associated with the movement of *nad5 *from its conserved position between *trnF *and *trnH *in *Katharina, Octopus, Haliotis*, and other caenogastropods, and with the break-up of *cox3*, *trnS1*, and *nad2 *from their conserved association within a block of genes including *cox3 *- 5 tRNAs - *nad3 - trnS1 - nad2 *in these same taxa. *TrnD *also shared a unique derived position between *cox1 *and *atp8 *in the Vermetidae. While *trnD *in *Haliotis *has differing neighbouring genes, the gene order *cox1*-*trnD *is shared with the chiton, *Katharina*, and *trnD-atp8 *is shared with *Octopus*, as well as all other caenogastropods. The derived vermetid arrangement thus appears to be associated with a switch in the position of *cox2 *relative to *cox1*, *trnD*, and *atp8*, along a lineage leading to the Vermetidae. In addition, the vermetids also shared a derived change in the relative positions of *trnY *and *trnM *within the cassette of 7 tRNA genes encoded on the "-" strand. Such distinctions between the mt genome of vermetids and other littorinimorphs are particularly relevant here given that there is both molecular and morphological support for the inclusion of littorines and vermetids in a clade of lower caenogastropods [28,37,38]. Gene rearrangements shared by vermetids to the exclusion of other Littorinimorpha (*Littorina*, *Oncomelania*, *Calyptraea*, and *Cymatium*), thus can be inferred to have occurred following the divergence of the ancestral vermetid from the common ancestor of these taxa.

Numerous gene order changes have also occurred within the family Vermetidae (Figure 5). The Vermetidae has a fossil record extending to the Late Cretaceous, suggesting that this gene reshuffling has happened within the past 65 million years, and based on molecular dating, perhaps within the past 38 million years [18]. Preliminary evidence suggests that the mt genome of *Dendropoma maximum *represents the ancestral gene order of the Vermetidae. The translocation of *trnK_1 _*and *trnP-nad6 *found in *Dendropoma gregarium *has also been shown to be a derived gene order change within this genus [18]. Relative to *D. maximum*, most gene rearrangements evident in the three other complete genomes have occurred between *trnR *and *trnT*. *Eualetes *differs from *D. maximum *in the position of four tRNAs: *trnA, trnP, trnL_UUR _and trnT*, with gene remolding events between *trnL_CUN _*and *trnL_UUR _*likely associated with gene order changes involving these two isoaccepting tRNA genes (see *Evidence for tRNA remolding and recruitment*, below). Differences between the gene arrangement of *Dendropoma maximum *and *Thylacodes *appear related to a gene duplication event in *Thylacodes *spanning *rrnS *to *nad1*, as inferred from gene vestiges of *rrnS*, *rrnL*, and *nad1 *and extra tRNA genes (*trnV*, *trnL_UUR_*). Details of this rearrangement will be examined further in another paper (Rawlings *et al*. in prep). Many of the conserved gene boundaries described above for the four complete vermetid genomes sequenced, even those shared with other caenogastropods, *Haliotis*, *Katharina*, and *Octopus*, were not evident in the partial genomes of *V. erectus *and *Thylaeodus *(Figures 1 &5). The scale of these rearrangements is impressive given that these changes have happened *within *a family of gastropods, and suggests that further sampling of the Vermetidae should uncover more changes and perhaps intermediate stages that may reveal the dynamics underlying the gene rearrangements shown here. The genome of *Thylacodes squamigerus *provides hope of this as vestiges of once functional genes discovered between annotated genes are helping to understand the mechanism of gene order change and the appearance of duplicated tRNAs. This expectation has also been borne out by additional data collected as part of a phylogenetic analysis of the Vermetidae: numerous gene order changes have now been uncovered within vermetid taxa based on short sequences (<3.5 kb) extending from *rrnS *to *nad1 *(Rawlings *et al*., in prep; data not shown). With further sampling of the Vermetidae, we anticipate that these gene order rearrangements will provide a suite of robust characters that can be used, in addition to morphological and nucleotide sequence characters, to build a well-supported phylogenetic hypothesis for this family and to firmly place the Vermetidae within the context of caenogastropod evolution.

### Evidence for tRNA remolding and recruitment

Implicit in the use of secondary structure characteristics and anticodon triplets to recognize tRNAs is the assumption that tRNA genes cannot change identity by simple nucleotide substitutions in their anticodon. For the most part this seems to be true within animal mt genomes, likely because of the presence of specific recognition elements that are required by tRNA synthetases to identify and correctly charge their associated tRNAs. However, evidence is accumulating that tRNAs do occasionally change identities. Through a process known as tRNA remolding, identity change does occur between the two isoaccepting leucine tRNA genes [25,26,28] and possibly also between the two isoaccepting serine tRNA genes [61]. Cases of tRNA identity change across non-isoaccepting tRNAs, referred to as tRNA recruitment, are now also coming to light [27]. The strongest evidence of tRNA remolding is the discovery of unexpectedly high levels of sequence similarity between two tRNA leucine genes [28]. Consequently, these dynamics are most easily recognized when they have happened recently, before mutational changes can obscure the common history of the duplicated tRNAs. Although gene remolding and recruitment events are often associated with changes in gene order, the pattern of duplicate loss may result in maintenance of the original gene order. In fact, recognizing gene remolding events can often help to uncover genome dynamics that are hidden at the level of gene order alone [28].

Given that leucine tRNAs seem particularly susceptible to remolding events [26,28], we investigated the sequence similarity between both leucine tRNAs within each vermetid taxon as well as other select caenogastropods, *Haliotis*, *Octopus*, and *Katharina *(Table 9). High sequence similarities (80% or greater) between leucine tRNA genes were found in five taxa (Table 9) suggesting that one tRNA has taken over the role of the other through a process of gene duplication, mutation in the anticodon triplet and the eventual loss of the original gene [26,28]. The *T. squamigerus *genome represents a particularly interesting case of this. Within this genome, the two copies of *L_UUR _*only share 67.6% sequence identity; in comparison there is 98.4% sequence similarity between *trnL_CUN _*and *trnL_UUR2_*. Consequently, we can assume that *trnL_UUR2 _*is a duplicated copy of *trnL_CUN _*that has subsequently undergone a mutation in the third position of the anticodon to assume the identity of *trnL_UUR_*. Such events argue for the exclusion of tRNA leucine genes in gene-order based phylogenetic analyses at high taxonomic levels; at low taxonomic levels, such as within the Vermetidae, however, they offer the promise of new phylogenetic characters and the discovery of gene dynamics that may not be evident at the level of gene order alone. Given the importance of identifying remolding events, it is surprising that so little attention is often paid to the dynamics of these two leucine tRNA genes. Failure to distinguish between these two genes correctly can also lead to mistakes in interpretation, where two genomes are considered to have identical gene orders, but differ in the position of these two leucine tRNAs (see comparisons between *Littorina saxatilis *and other caenogastropods in [15,48,53]).

**Table 9:** Percentage similarity in nucleotide sequences between isoaccepting leucine tRNA genes (*trnL_UUR _*and *trnL_CUN_*) within the mt genomes of six vermetids, sixteen additional caenogastropods, and three other molluscs for comparison

Species	tRNA comparison^ab^	Percent similarity^c^
*Dendropoma maximum*	*L_UUR_-L_CUN_*	60.9%
*Dendropoma gregarium*	*L_UUR_-L_CUN_*	69.0%
*Eualetes tulipa*	*L_CUN_-/-L_UUR_*	**98.4%**
*Thylacodes squamigerus*^d^	*L_CUN_-/-L_UUR1_*	66.2%
	*L_CUN_-/-L_UUR2_*	**98.4%**
	*L_UUR1_-L_UUR2_*	67.6%
*Vermetus erectus*	*L_UUR_-L_CUN_*	76.6%
*Thylaeodus sp*.	*L_CUN_-/-L_UUR_*	**87.0%**
*Oncomelania*	*L_UUR_-L_CUN_*	**91.2%**
*Littorina*	*L_UUR_-L_CUN_*	**80.3%**
*Calyptraea*	*L_CUN_-L_UUR_*	58.0%
*Cymatium*	*L_CUN_-L_UUR_*	61.8%
*Thais*	*L_CUN_-L_UUR_*	58.0%
*Rapana*	*L_CUN_-L_UUR_*	Insufficient data^e^
*Conus textile*	*L_CUN_-L_UUR_*	60.9%
*Conus borgesi*	*L_CUN_-L_UUR_*	60.9%
*Lophiotoma*	*L_CUN_-L_UUR_*	62.3%
*Ilyanassa*	*L_CUN_-L_UUR_*	64.7%
*Nassarius*	*L_CUN_-L_UUR_*	64.7%
*Bolinus*	*L_CUN_-L_UUR_*	62.3%
*Cancellaria*	*L_CUN_-L_UUR_*	52.7%
*Cymbium*	*L_CUN_-L_UUR_*	58.2%
*Fusiturris*	*L_CUN_-L_UUR_*	66.7%
*Terebra*	*L_CUN_-L_UUR_*	59.4%
*Haliotis rubra*	*L_CUN_-L_UUR_*	64.2%
*Katharina tunicata*	*L_CUN_-L_UUR_*	48.5%
*Octopus vulgaris*	*L_CUN_-L_UUR_*	66.7%

^a ^tRNA genes were aligned using Clustal X with subsequent adjustments made according to secondary structure.

^b ^Leucine tRNAs are presented according to relative order and position: one dash indicates that the two tRNAs are adjacent to each other; two dashes separated by a backslash indicate that these two genes are separated by at least one other gene.

^c ^Percent similarity reflects the number of nucleotide matches over the total length of the alignment, excluding the third base of the anticodon triplet.

^d ^Because *Thylacodes *had two copies of *trnL_UUR_*, three pairwise comparisons between leucine tRNA genes were made for this species.

^e ^Sequence contained ambiguous nucleotides.

**Figure 6:**
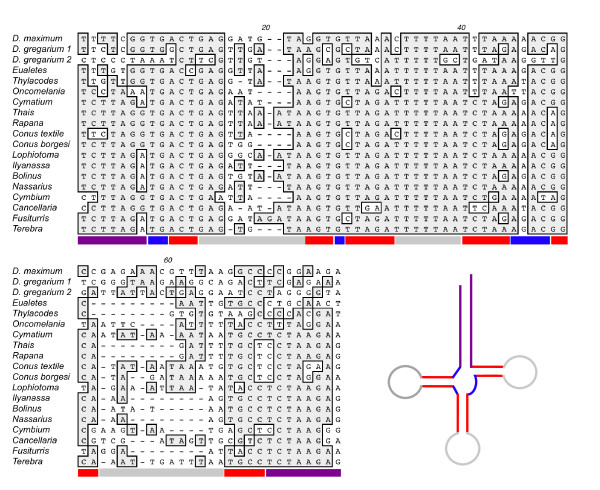
**Structural alignments of lysine tRNA genes sampled from four vermetid gastropods and other select caenogastropods**. Two tRNA genes are included from *Dendropoma gregarium*, one located between *trnV *and *trnP *(*trnK1*), the other located between *trnI *and *nad3 *(*trnK2*). Nucleotides appearing in >50% of taxa at a given position in the gene are shaded grey. Colors below each region of the alignment are used to show the associated position in the colored tRNA secondary structure model.

The presence of a second *trnK *within the genome of *Dendropoma gregarium *is suggestive of a past duplication event within this genome, likely the result of slipped-strand mispairing during replication. The sequence similarity between the two lysine tRNAs (*trnK*) is not high (38.0%). Comparisons between these sequences and the sequences of presumed *trnK *orthologs from other taxa revealed strong similarities between these and the *trnK_1 _*located between *trnV *and *trnP *(Figure 6). The second *trnK *(*trnK_2_*), located between *trnI *and *nad3 *lacked many of the conserved sequence elements present in other *trnK*s (Figure 6). We compared *trnK_2 _*with other tRNAs within the genome of *D. gregarium *to determine if this might represent a case of tRNA recruitment [27], but did not uncover any strong matches with any other gene. Consequently, the origin and function of this putative tRNA are currently unclear.

## Conclusions

Here we have presented gene order and sequence characteristics for six gastropods within the family Vermetidae, including the complete mt genomes of four species (*Dendropoma maximum*, *D. gregarium*, *Eualetes tulipa*, and *Thylacodes squamigerus*) and the partial mt genomes of two others (*Vermetus erectus *and *Thylaeodus sp*.). The publication of these genomes increases the number of complete caenogastropod mt genomes sequenced from 16 to 20 and presents the first direct evidence of major gene order rearrangements within the Littorinimorpha.

While molluscs have long been recognized as having unusually dynamic mt genomes, preliminary sampling of the Caenogastropoda represented by genomes from 16 species has suggested a model of highly conserved mt gene order. Our results reverse this trend and provide further evidence of the lability of the molluscan mt genome by describing extensive gene order changes that have occurred within one family of caenogastropod molluscs, the Vermetidae. Based on these results, we anticipate that the mt genomes of caenogastropods exhibit a variety of gene order arrangements, which, if explored and exploited, could provide a wealth of phylogenetically-informative characters to enhance our understanding of the evolutionary radiation of this diverse clade of gastropods.

Despite the extent of mt gene rearrangement within the Vermetidae, their genomes exhibit similar characteristics to other caenogastropods. Each complete genome contained the full complement of 37 genes, although additional tRNA genes were evident in the mt genomes of *D. gregarium *(*trnK*) and *Thylacodes squamigerus *(*trnV*, *trnL_UUR_*). Protein-encoding and rRNA genes were all encoded on the same strand (i.e. the "+" strand), not unusual for caenogastropods, whereas tRNA genes, the most mobile mt genomic components, were distributed between the "+" and "-" strands. Nucleotide skews patterns (i.e. AT and GC-skews) of vermetid genomes and their various components were similar to those described for other caenogastropods, although the AT bias was less pronounced than other caenogastropods described to date. Although no control regions were definitively identified, a compelling trend in the relationship between nucleotide composition and position from *nad1 *was uncovered, which appears worthy of further investigation.

Our results also demonstrate that focused sampling of mt genomes at low taxonomic levels can be extremely productive, both in terms of uncovering characters useful in phylogeny and in understanding more fully the evolutionary dynamics and mechanics of mt gene rearrangements. Each of the six vermetid mt genomes examined had a unique gene order, with evidence of gene rearrangements involving translocations of tRNA and protein-encoding genes, and one gene inversion, occurring within this family. Additional rearrangements have also been uncovered amongst other representatives of this family suggesting that further sampling of complete mt genomes within the Vermetidae will also prove useful. The extent of gene rearrangement within such an evolutionarily young group offers the opportunity to explore gene dynamics such as tRNA remolding, which appears to have been rife within this family, and to investigate its consequences for phylogenetic analyses based on gene orders. The sampling of mt genomes, such as that of *Thylacodes squamigerus*, in the intermediate stages of a gene rearrangement, may also help to illuminate mechanisms of gene translocation and inversion and to interpret past events that have shaped an organism's current mt gene order. Continued studies of the mt genome dynamics within the Vermetidae will improve our understanding of this family's phylogeny, its phylogenetic placement within the Littorinimorpha, and the mechanisms and processes that have acted to shape the mt genomes of this family and other metazoans.

## Authors' contributions

RB and TAR obtained the samples. TAR and TMC extracted the DNA and amplified the genomes which were then sequenced by JLB. TAR and MJM annotated the genome, MJM undertook the descriptive analyses and TAR and MJM wrote the first draft of the manuscript. All authors participated in subsequent revisions of the manuscript.

## Supplementary Material

Additional file 1 **Figure S1**. Inferred tRNA secondary structures based on the nucleotide sequences of 22 mitochondrial tRNA genes identified from the complete mt genome of ***Dendropoma maximum***. tRNA genes are labeled according to their amino acid specificity and are arranged alphabetically. Click here for file

Additional file 2 **Figure S2**. Inferred tRNA secondary structures based on the nucleotide sequences of 23 mitochondrial tRNA genes identified from the complete mt genome of ***Dendropoma gregarium***. tRNA genes are labeled according to their amino acid specificity and are arranged alphabetically. Click here for file

Additional file 3 **Figure S3**. Inferred tRNA secondary structures based on the nucleotide sequences of 22 mitochondrial tRNA genes identified from the complete mt genome of ***Eualetes tulipa***. tRNA genes are labeled according to their amino acid specificity and are arranged alphabetically. Click here for file

Additional file 4 **Figure S4**. Inferred tRNA secondary structures based on the nucleotide sequences of 24 mitochondrial tRNA genes identified from the complete mt genome of ***Thylacodes squamigerus***. tRNA genes are labeled according to the amino acid specificity and are arranged alphabetically. Click here for file

Additional file 5 **Figure S5**. Inferred tRNA secondary structures based on the nucleotide sequences of eight mitochondrial tRNA genes identified from the partial mt genome of ***Thylaeodus sp***. tRNA genes are labeled according to their amino acid specificity and are arranged alphabetically. Click here for file

Additional file 6 **Figure S6**. Inferred tRNA secondary structures based on the nucleotide sequences of five mitochondrial tRNA genes identified from the partial mt genome of ***Vermetus erectus***. tRNA genes are labeled according to their amino acid specificity and are arranged alphabetically. Click here for file

Additional file 7 **Table S1**. Base compositions and nucleotide skews for new vermetid mt genomes, existing caenogastropod mt genomes, and other select molluscs. Click here for file

Additional file 8 **Table S2**. The conservation of amino acid identity in select codons from ***cox1***, ***cox2***, and ***cox3 ***genes from the mt genomes of ***Dendropoma maximum***, ***D. gregarium***, ***Eualetes tulipa***, and ***Thylacodes squamigerus***. Click here for file

Additional file 9 **Table S3**. Summary of codon usage across all protein-encoding genes in the mitochondrial genomes of ***Dendropoma maximum***, ***D. gregarium***, ***Eualetes tulipa***, and ***Thylacodes squamigerus***. Click here for file
