# Supplementary figures and images for: Sessile snails, dynamic genomes: gene rearrangements within the mitochondrial genome of a family of caenogastropod molluscs
Source: BMC Genomics. 2010 Jul 19;11:440. doi: 10.1186/1471-2164-11-440 (PMC3091637; doi:10.1186/1471-2164-11-440)

Figure S1

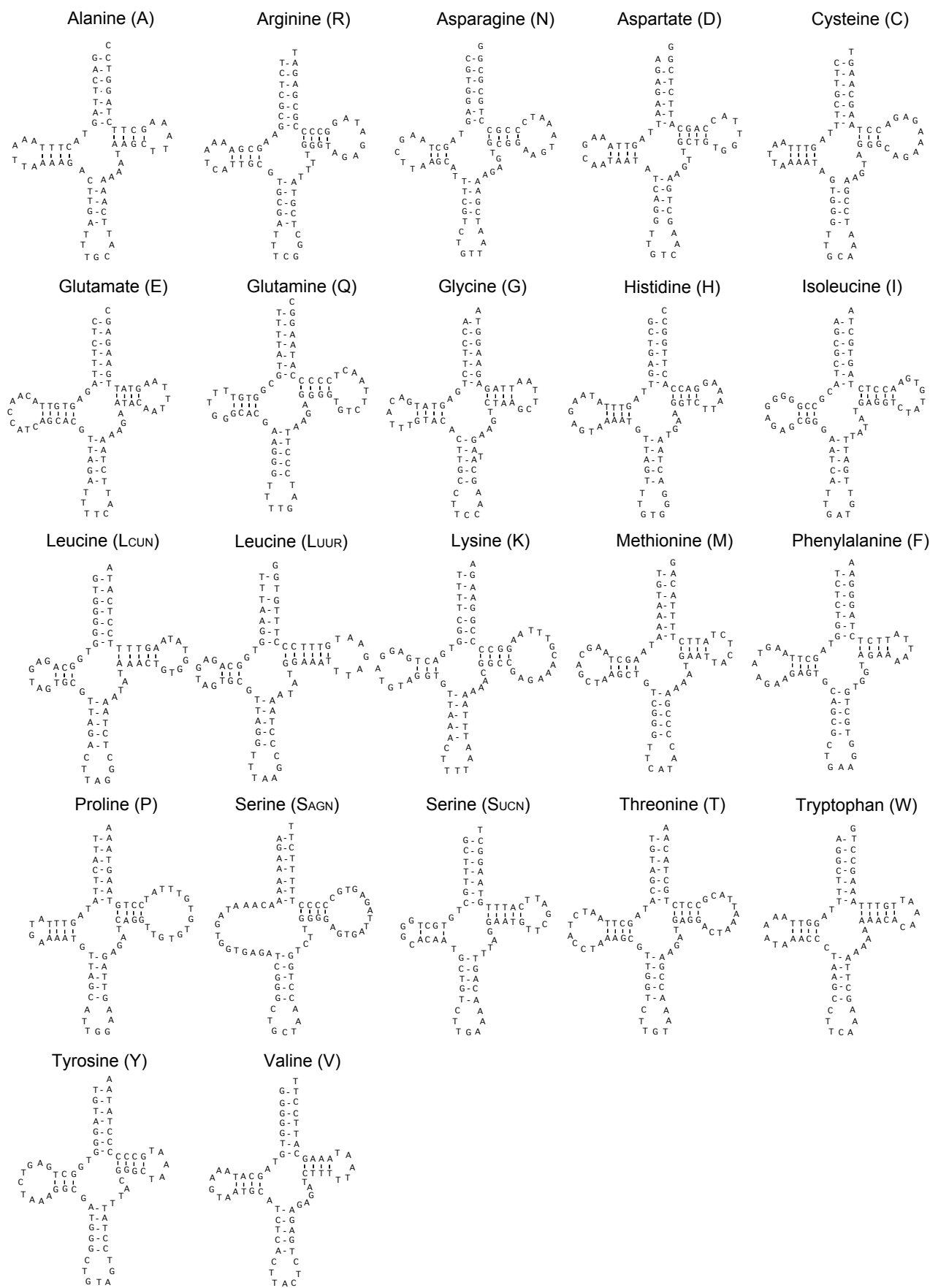

Supplement: Additional file 1 — Figure S1. Inferred tRNA secondary structures based on the nucleotide sequences of 22 mitochondrial tRNA genes identified from the complete mt genome of Dendropoma maximum. tRNA genes are labeled according to their amino acid specificity and are arranged alphabetically. [file 1471-2164-11-440-S1.PDF]

Figure S2

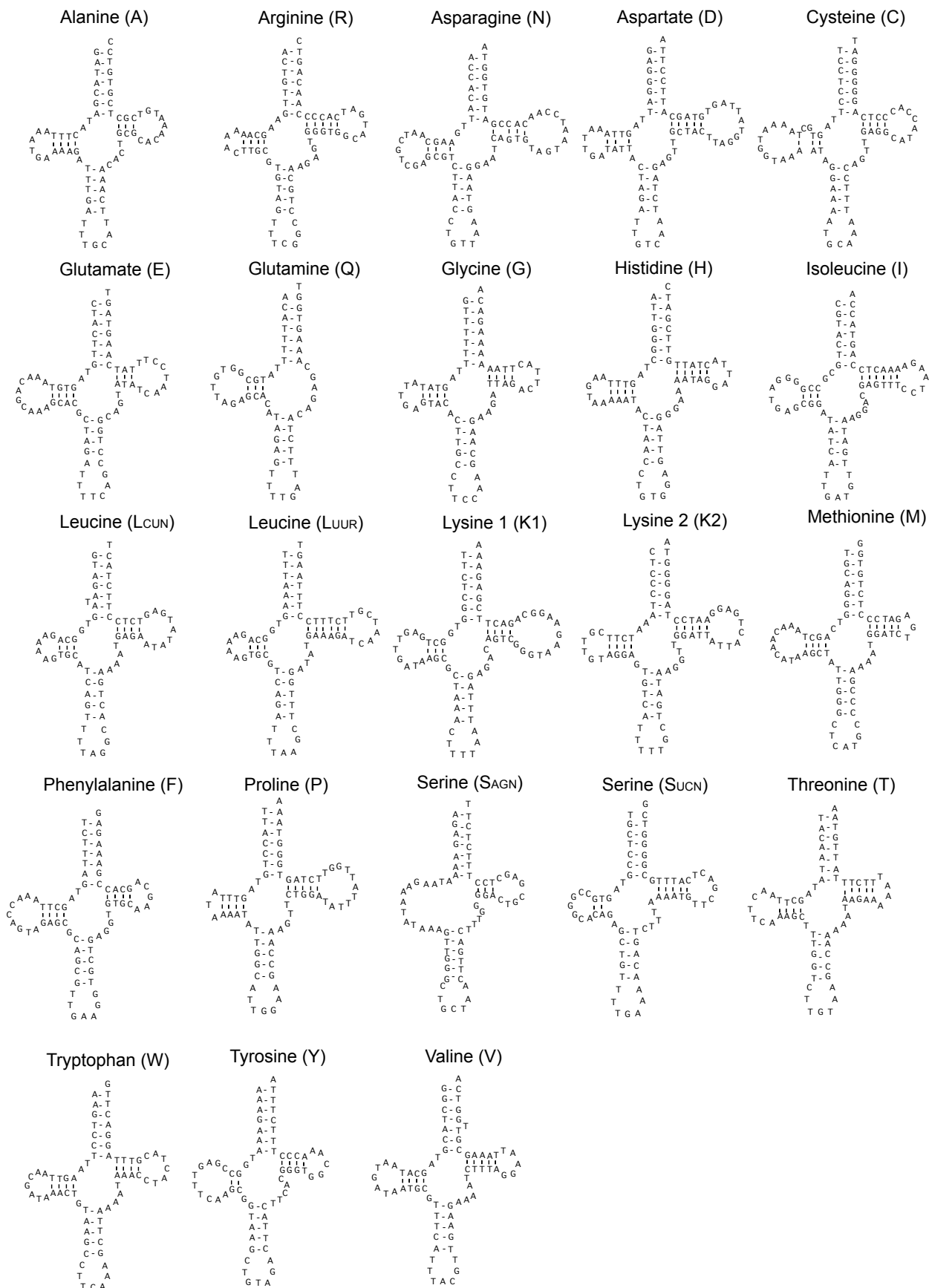

Supplement: Additional file 2 — Figure S2. Inferred tRNA secondary structures based on the nucleotide sequences of 23 mitochondrial tRNA genes identified from the complete mt genome of Dendropoma gregarium. tRNA genes are labeled according to their amino acid specificity and are arranged alphabetically. [file 1471-2164-11-440-S2.PDF]

Figure S3

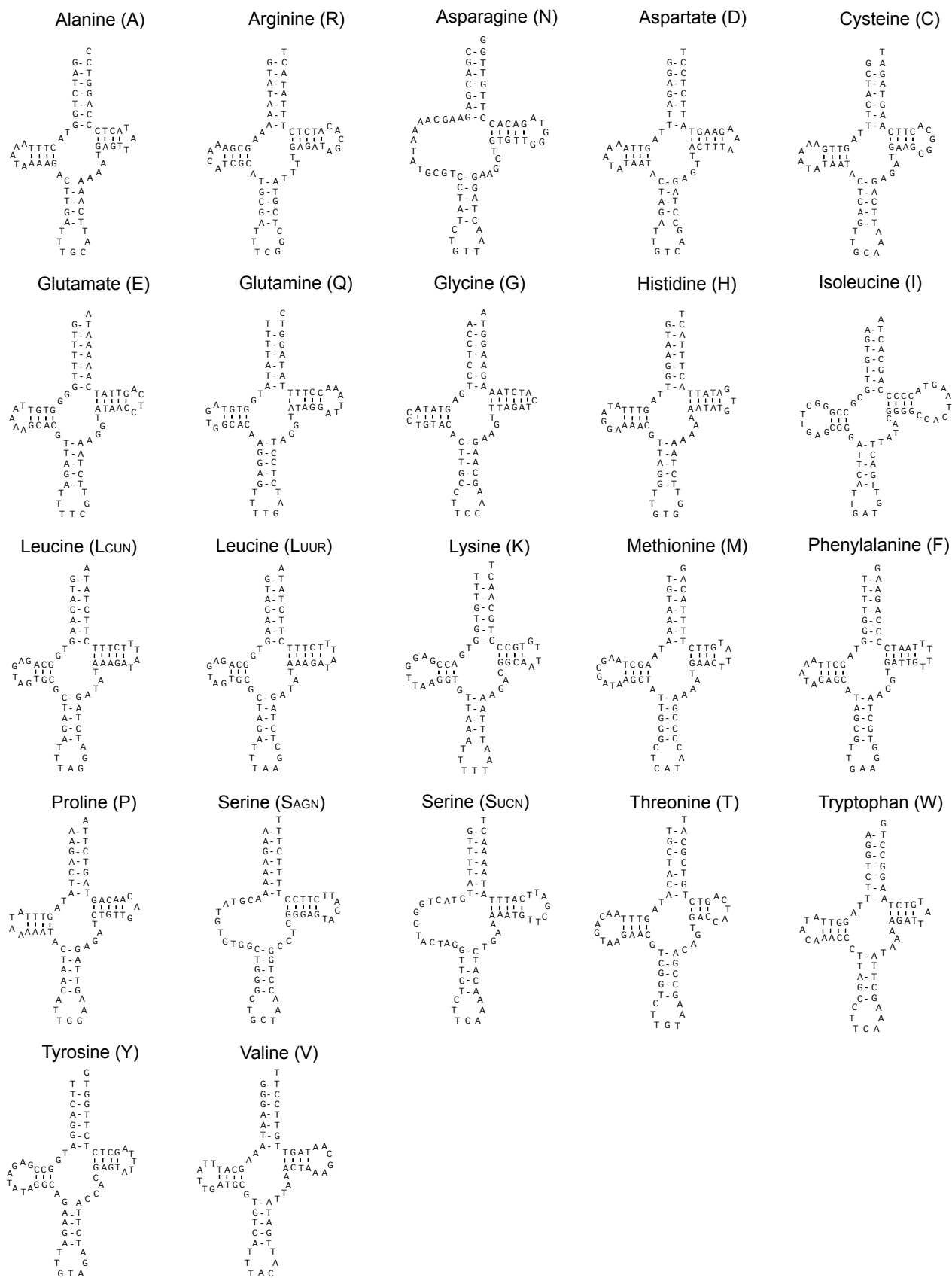

Supplement: Additional file 3 — Figure S3. Inferred tRNA secondary structures based on the nucleotide sequences of 22 mitochondrial tRNA genes identified from the complete mt genome of Eualetes tulipa. tRNA genes are labeled according to their amino acid specificity and are arranged alphabetically. [file 1471-2164-11-440-S3.PDF]

Figure S4

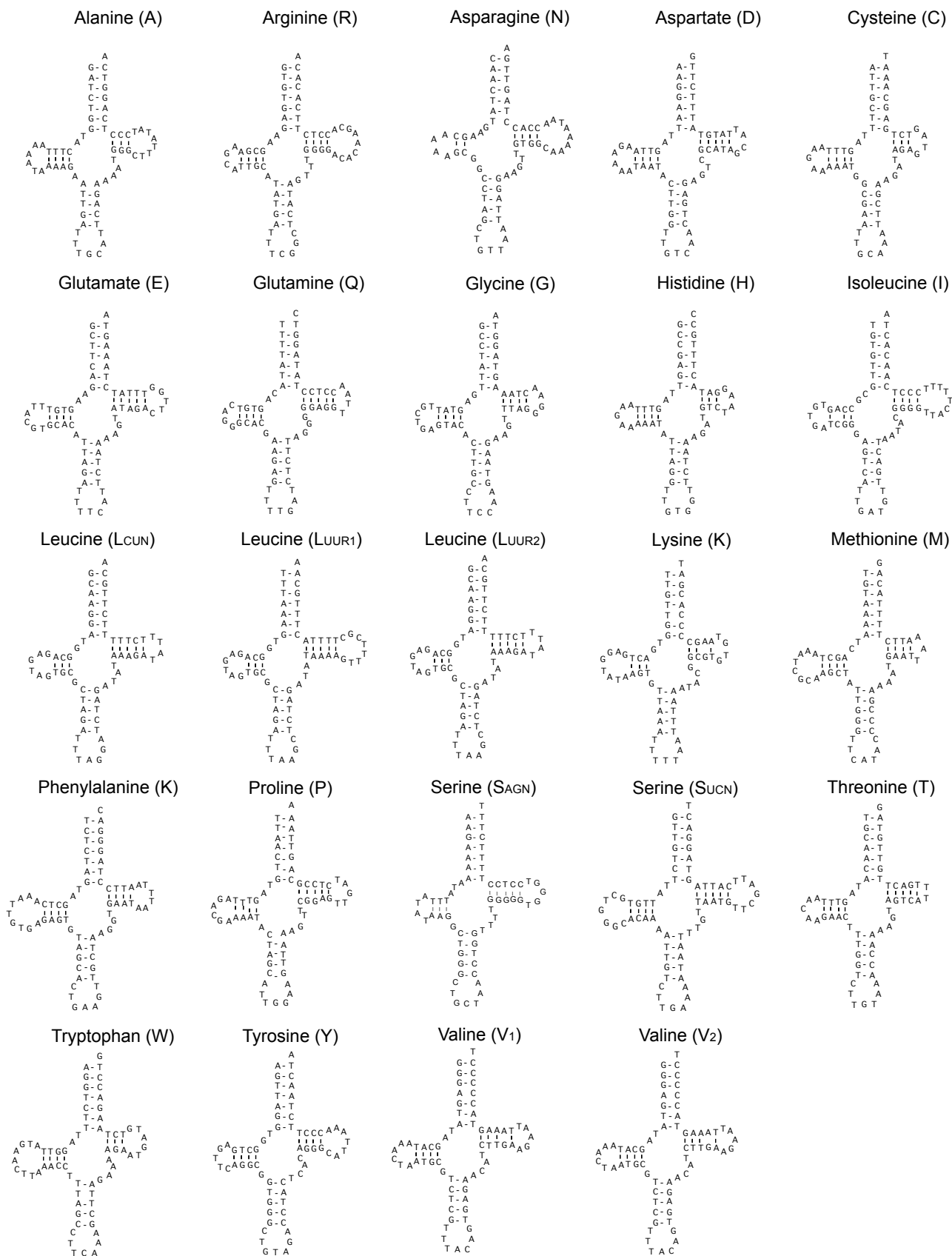

Supplement: Additional file 4 — Figure S4. Inferred tRNA secondary structures based on the nucleotide sequences of 24 mitochondrial tRNA genes identified from the complete mt genome of Thylacodes squamigerus. tRNA genes are labeled according to the amino acid specificity and are arranged alphabetically. [file 1471-2164-11-440-S4.PDF]

Figure S5

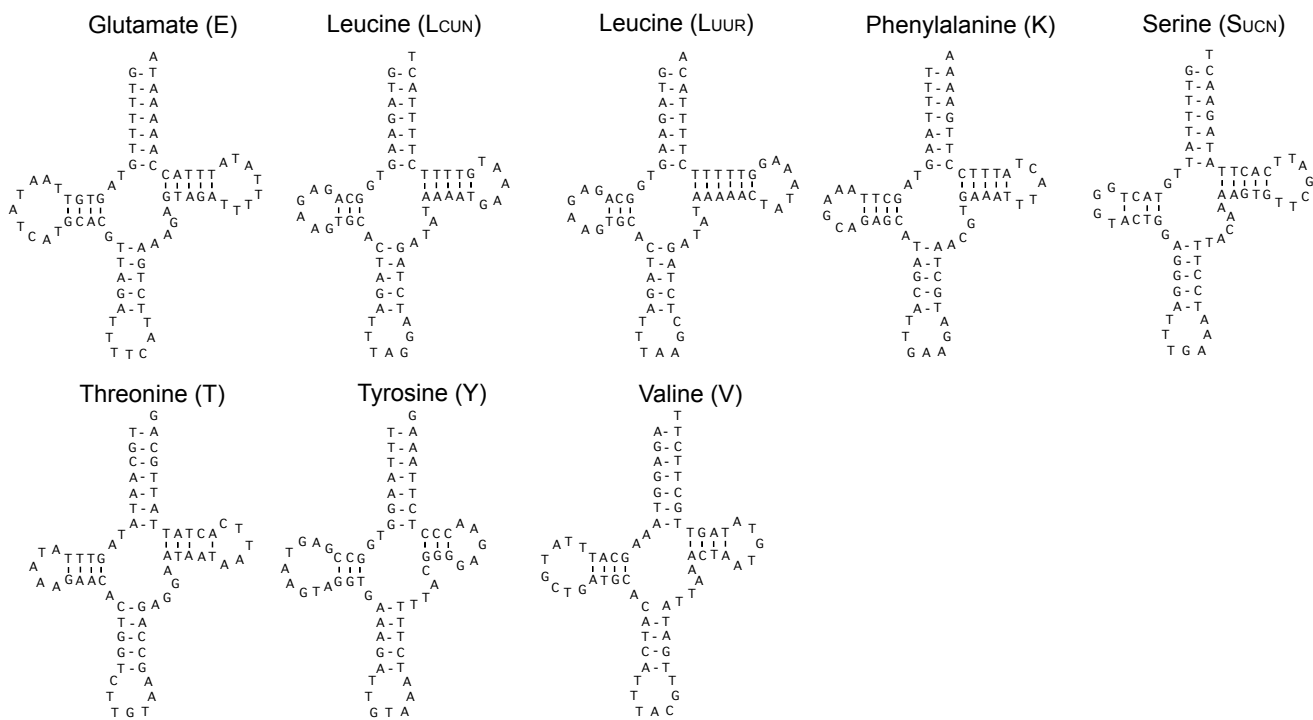

Supplement: Additional file 5 — Figure S5. Inferred tRNA secondary structures based on the nucleotide sequences of eight mitochondrial tRNA genes identified from the partial mt genome of Thylaeodus sp. tRNA genes are labeled according to their amino acid specificity and are arranged alphabetically. [file 1471-2164-11-440-S5.PDF]

Figure S6

Histidine (H)

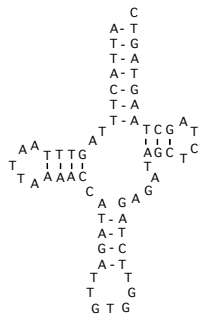

Isoleucine (I)

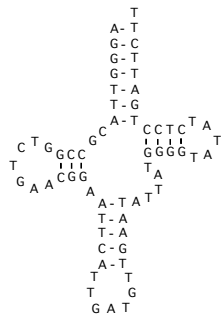

Leucine (L<sub>CUN</sub>)

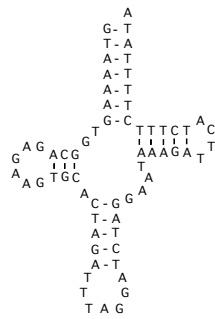

Leucine (L<sub>UUR</sub>)

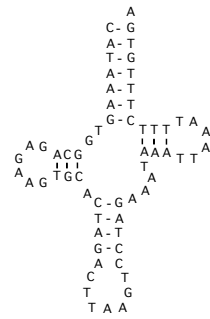

Valine (V)

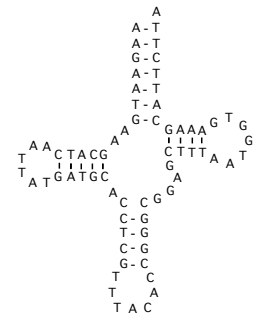

Supplement: Additional file 6 — Figure S6. Inferred tRNA secondary structures based on the nucleotide sequences of five mitochondrial tRNA genes identified from the partial mt genome of Vermetus erectus. tRNA genes are labeled according to their amino acid specificity and are arranged alphabetically. [file 1471-2164-11-440-S6.PDF]
