# Supplementary material for: Sessile snails, dynamic genomes: gene rearrangements within the mitochondrial genome of a family of caenogastropod molluscs
Source: BMC Genomics. 2010 Jul 19;11:440. doi: 10.1186/1471-2164-11-440 (PMC3091637; doi:10.1186/1471-2164-11-440)
Supplement: Additional file 7 — Table S1. Base compositions and nucleotide skews for new vermetid mt genomes, existing caenogastropod mt genomes, and other select molluscs. [file 1471-2164-11-440-S7.PDF]

Table S1: Base compositions and nucleotide skews for new vermetid mt genomes, existing caenogastropod mt genomes, and other select molluscs.

	T (%)[a]	C (%)[a]	A (%)[a]	G (%)[a]	N[b]	Total bp	%AT	%CG	AT Skew[c]	GC Skew[c]
Dendropoma maximum	5726 (36.8)	2368 (15.2)	3527 (22.6)	3953 (25.4)	4	15578	59.4	40.6	-0.238	0.251
Dendropoma gregarium	5575 (35.6)	2675 (17.1)	3870 (24.7)	3521 (22.5)	0	15641	60.4	39.6	-0.181	0.137
Eualetes tulipa	5376 (35.7)	2333 (15.5)	3990 (26.5)	3379 (22.4)	0	15078	62.1	37.9	-0.148	0.183
Thylacodes squamigerus	5445 (35.0)	2857 (18.4)	3986 (25.6)	3255 (20.9)	1	15544	60.7	39.3	-0.155	0.065
Vermetus erectus[d]	1652 (35.0)	720 (15.3)	1378 (29.2)	968 (20.5)	0	4718	64.2	35.8	-0.090	0.147
Thylaeodus sp.[d]	2372 (37.0)	915 (14.3)	1898 (29.6)	1230 (19.2)	0	6415	66.6	33.4	-0.111	0.147
Oncomelania hupensis	5669 (37.3)	2424 (16.0)	4552 (30.0)	2537 (16.7)	0	15182	67.3	32.7	-0.109	0.023
Littorina saxatilis[d]	2922 (36.4)	1423 (17.7)	2449 (30.5)	1228 (15.3)	0	8022	67.0	33.0	-0.088	-0.074
Calyptraea chinensis [d]	3607 (42.3)	968 (11.3)	2625 (30.8)	1330 (15.6)	0	8530	73.1	26.9	-0.158	0.158
Cymatium parthenopeum	5829 (38.2)	2279 (14.9)	4724 (30.9)	2438 (16.0)	0	15270	69.1	30.9	-0.105	0.034
Thais clavigera	5767 (37.7)	2479 (16.2)	4350 (28.5)	2689 (17.6)	0	15285	66.2	33.8	-0.140	0.041
Rapana venosa	5944 (38.9)	2267 (14.8)	4560 (29.9)	2449 (16.0)	52	15272	68.8	30.9	-0.132	0.039
Ilyanassa obsoleta	5925 (38.8)	2272 (14.9)	4586 (30.1)	2473 (16.2)	7	15263	68.9	31.1	-0.127	0.042
Nassarius reticulatus	5746 (37.6)	2388 (15.6)	4555 (29.8)	2582 (16.9)	0	15271	67.5	32.5	-0.116	0.039
Bolinus brandaris	5873 (38.2)	2337 (15.2)	4472 (29.1)	2698 (17.5)	0	15380	67.3	32.7	-0.135	0.072
Cancellaria cancellata	6964 (41.8)	1978 (11.9)	4714 (28.3)	2992 (18.0)	0	16648	70.1	29.9	-0.193	0.204
Conus textile	5906 (38.0)	2436 (15.7)	4239 (27.2)	2981 (19.2)	0	15562	65.2	34.8	-0.164	0.101
Conus borgesi	5980 (38.5)	2269 (14.6)	4453 (28.7)	2834 (18.2)	0	15536	67.2	32.8	-0.146	0.111

Lophiotoma cerithiformis	5852 (38.0)	2395 (15.6)	4588 (29.8)	2545 (16.6)	0	15380	67.9	32.1	-0.121	0.030
Fusiturris similis	5816 (37.3)	2509 (16.1)	4534 (29.1)	2736 (17.5)	0	15595	66.4	33.6	-0.124	0.043
Terebra dimidiata	6608 (40.0)	2084 (12.6)	4233 (25.6)	3588 (21.7)	0	16513	65.7	34.3	-0.219	0.265
Cymbium olla	5936 (38.6)	2166 (14.1)	4607 (30.0)	2666 (17.3)	0	15375	68.6	31.4	-0.126	0.103
Haliotis rubra	4150 (24.5)	4515 (26.7)	5844 (34.6)	2398 (14.2)	0	16907	59.1	40.9	0.170	-0.306
Katharina tunicata	5905 (38.0)	1847 (11.9)	4876 (31.4)	2891 (18.6)	13	15532	69.4	30.5	-0.095	0.220
Octopus vulgaris	5309 (33.7)	2764 (17.6)	6478 (41.1)	1193 (7.6)	0	15744	74.9	25.1	0.099	-0.397

[a] Base compositions are reported as total numbers of each nucleotide and % of total number of nucleotides within each genome.
[b] "N" refers to ambiguous nucleotides.
[c] Genome-level AT and GC skews were calculated according to [44].
[d] Only partial mt genomes were available.
