# Supplementary material for: Sessile snails, dynamic genomes: gene rearrangements within the mitochondrial genome of a family of caenogastropod molluscs
Source: BMC Genomics. 2010 Jul 19;11:440. doi: 10.1186/1471-2164-11-440 (PMC3091637; doi:10.1186/1471-2164-11-440)
Supplement: Additional file 8 — Table S2. The conservation of amino acid identity in select codons from cox1, cox2, and cox3 genes from the mt genomes of Dendropoma maximum, D. gregarium, Eualetes tulipa, and Thylacodes squamigerus. [file 1471-2164-11-440-S8.PDF]

Table S2. The conservation of amino acid identity in select codons from *cox1*, *cox2*, and *cox3* genes from the mt genomes of *Dendropoma maximum*, *D. gregarium*, *Eualetes tulipa*, and *Thylacodes squamigerus*.

		cox1			*cox2*			*cox3*			Total		
	Codon[a]	Conserved[b]	All[c]	Percent	Conserved[b]	All[c]	Percent	Conserved[b]	All[c]	Percent	Conserved[b]	All[c]	Percent
Dendropoma maximum	TGA	10	10	100.0%	5	6	83.3%	7	7	100.0%	22	23	95.7%
	ATA	15	17	88.2%	4	6	66.7%	4	4	100.0%	23	27	85.2%
	AGA	1	2	50.0%	0	0	n/a	2	3	66.7%	3	5	60.0%
	AGG	2	2	100.0%	0	0	n/a	2	2	100.0%	4	4	100.0%
	AAA	3	4	75.0%	1	3	33.3%	0	1	0.0%	4	8	50.0%
Dendropoma gregarium	TGA	9	9	100.0%	4	4	100.0%	8	9	88.9%	21	22	95.5%
	ATA	9	9	100.0%	5	5	100.0%	2	2	100.0%	16	16	100.0%
	AGA	0	1	0.0%	0	1	0.0%	4	7	57.1%	4	9	44.4%
	AGG	2	2	100.0%	0	1	0.0%	0	1	0.0%	2	4	50.0%
	AAA	4	4	100.0%	0	0	n/a	0	0	n/a	4	4	100.0%
Eualetes tulipa	TGA	12	12	100.0%	4	4	100.0%	10	10	100.0%	26	26	100.0%
	ATA	14	17	82.4%	3	7	42.9%	1	4	25.0%	18	28	64.3%
	AGA	0	0	n/a	1	2	50.0%	3	4	75.0%	4	6	66.7%
	AGG	1	1	100.0%	1	3	33.3%	1	1	100.0%	3	5	60.0%
	AAA	5	5	100.0%	2	2	100.0%	1	3	33.3%	8	10	80.0%
Thylacodes squamigerus	TGA	9	9	100.0%	3	3	100.0%	5	6	83.3%	17	18	94.4%
	ATA	18	21	85.7%	5	7	71.4%	3	6	50.0%	26	34	76.5%
	AGA	0	0	n/a	1	2	50.0%	2	2	100.0%	3	4	75.0%
	AGG	2	2	100.0%	1	2	50.0%	0	1	0.0%	3	5	60.0%
	AAA	3	3	100.0%	2	3	66.7%	1	1	100.0%	6	7	85.7%

[a] Codons whose amino acid identity has changed in the mt genetic code of other metazoans [46] .

[b] The number of codons whose corresponding amino acid occurred in a conserved position in comparison with other select caenogastropods (>50%).

[c] The total number of codons within the gene.
