# Supplementary material for: Sessile snails, dynamic genomes: gene rearrangements within the mitochondrial genome of a family of caenogastropod molluscs
Source: BMC Genomics. 2010 Jul 19;11:440. doi: 10.1186/1471-2164-11-440 (PMC3091637; doi:10.1186/1471-2164-11-440)
Supplement: Additional file 9 — Table S3. Summary of codon usage across all protein-encoding genes in the mitochondrial genomes of Dendropoma maximum, D. gregarium, Eualetes tulipa, and Thylacodes squamigerus. [file 1471-2164-11-440-S9.PDF]

Table S3. Summary of codon usage across all protein-encoding genes in the mitochondrial genomes of *D. maximum*, *D. gregarium*, *E. tulipa*, and *T. squamigerus*.

Codon	AA	*D. maximum*			*D. gregarium*			*E. tulipa*			*T. squamigerus*		
		Total #	Total %	% Amino acid	Total #	Total %	% Amino acid	Total #	Total %	% Amino acid	Total #	Total %	% Amino acid
TTT	F	241	6.5	81.1	197	5.3	71.4	219	5.9	77.7	229	6.2	75.6
TTC	F	56	1.5	18.9	79	2.1	28.6	63	1.7	22.3	74	2.0	24.4
TTA	L	175	4.7	45.5	212	5.7	60.1	218	5.9	62.1	183	4.9	67.8
TTG	L	210	5.7	54.5	141	3.8	39.9	133	3.6	37.9	87	2.3	32.2
TCT	S	114	3.1	58.8	88	2.4	44.9	81	2.2	41.8	77	2.1	38.5
TCC	S	20	0.5	10.3	41	1.1	20.9	21	0.6	10.8	41	1.1	20.5
TCA	S	33	0.9	17.0	47	1.3	24.0	56	1.5	28.9	49	1.3	24.5
TCG	S	27	0.7	13.9	20	0.5	10.2	36	1.0	18.6	33	0.9	16.5
TAT	Y	112	3.0	88.2	108	2.9	70.1	119	3.2	71.7	91	2.5	61.5
TAC	Y	15	0.4	11.8	46	1.2	29.9	47	1.3	28.3	57	1.5	38.5
TAA	*	3	0.1	30.0	5	0.1	50.0	2	0.1	22.2	4	0.1	36.4
TAG	*	7	0.2	70.0	5	0.1	50.0	7	0.2	77.8	7	0.2	63.6
TGT	C	49	1.3	83.1	49	1.3	69.0	49	1.3	79.0	29	0.8	54.7
TGC	C	10	0.3	16.9	22	0.6	31.0	13	0.3	21.0	24	0.6	45.3
TGA	W	55	1.5	49.5	60	1.6	52.6	75	2.0	67.6	54	1.5	52.9
TGG	W	56	1.5	50.5	54	1.5	47.4	36	1.0	32.4	48	1.3	47.1
CTT	L	127	3.4	55.5	85	2.3	30.7	73	2.0	29.3	105	2.8	32.1
CTC	L	26	0.7	11.4	44	1.2	15.9	19	0.5	7.6	40	1.1	12.2
CTA	L	30	0.8	13.1	90	2.4	32.5	96	2.6	38.6	107	2.9	32.7
CTG	L	46	1.2	20.1	58	1.6	20.9	61	1.6	24.5	75	2.0	22.9
CCT	P	74	2.0	51.0	57	1.5	39.9	71	1.9	51.1	57	1.5	40.7
CCC	P	21	0.6	14.5	32	0.9	22.4	23	0.6	16.5	36	1.0	25.7
CCA	P	23	0.6	15.9	28	0.8	19.6	24	0.6	17.3	24	0.6	17.1
CCG	P	27	0.7	18.6	26	0.7	18.2	21	0.6	15.1	23	0.6	16.4
CAT	H	59	1.6	75.6	57	1.5	69.5	54	1.5	64.3	49	1.3	57.0
CAC	H	19	0.5	24.4	25	0.7	30.5	30	0.8	35.7	37	1.0	43.0
CAA	Q	19	0.5	27.5	31	0.8	46.3	29	0.8	46.0	36	1.0	51.4
CAG	Q	50	1.4	72.5	36	1.0	53.7	34	0.9	54.0	34	0.9	48.6
CGT	R	15	0.4	22.1	17	0.5	23.3	17	0.5	24.6	11	0.3	16.9
CGC	R	5	0.1	7.4	8	0.2	11.0	10	0.3	14.5	6	0.2	9.2
CGA	R	27	0.7	39.7	28	0.8	38.4	19	0.5	27.5	26	0.7	40.0
CGG	R	21	0.6	30.9	20	0.5	27.4	23	0.6	33.3	22	0.6	33.8
ATT	I	175	4.7	85.0	174	4.7	77.3	149	4.0	79.3	174	4.7	72.5
ATC	I	31	0.8	15.0	51	1.4	22.7	39	1.0	20.7	66	1.8	27.5
ATA	M	71	1.9	46.4	96	2.6	55.2	124	3.3	56.1	97	2.6	59.1
ATG	M	82	2.2	53.6	78	2.1	44.8	97	2.6	43.9	67	1.8	40.9
ACT	T	71	1.9	47.3	71	1.9	41.8	63	1.7	36.2	85	2.3	50.0
ACC	T	14	0.4	9.3	28	0.8	16.5	22	0.6	12.6	25	0.7	14.7
ACA	T	33	0.9	22.0	41	1.1	24.1	65	1.7	37.4	36	1.0	21.2
ACG	T	32	0.9	21.3	30	0.8	17.6	24	0.6	13.8	24	0.6	14.1
AAT	N	78	2.1	78.0	70	1.9	73.7	79	2.1	82.3	74	2.0	65.5
AAC	N	22	0.6	22.0	25	0.7	26.3	17	0.5	17.7	39	1.1	34.5
AAA	K	36	1.0	43.9	39	1.0	52.7	42	1.1	56.8	38	1.0	46.9
AAG	K	46	1.2	56.1	35	0.9	47.3	32	0.9	43.2	43	1.2	53.1
AGT	S	50	1.4	34.2	41	1.1	28.9	54	1.5	32.0	44	1.2	29.1
AGC	S	12	0.3	8.2	26	0.7	18.3	20	0.5	11.8	29	0.8	19.2
AGA	S	41	1.1	28.1	40	1.1	28.2	51	1.4	30.2	48	1.3	31.8
AGG	S	43	1.2	29.5	35	0.9	24.6	44	1.2	26.0	30	0.8	19.9
GTT	V	144	3.9	42.2	94	2.5	29.5	90	2.4	27.4	113	3.0	37.8
GTC	V	19	0.5	5.6	47	1.3	14.7	13	0.3	4.0	50	1.3	16.7
GTA	V	65	1.8	19.1	93	2.5	29.2	124	3.3	37.8	81	2.2	27.1
GTG	V	113	3.1	33.1	85	2.3	26.6	101	2.7	30.8	55	1.5	18.4
GCT	A	149	4.0	53.2	93	2.5	39.2	101	2.7	41.6	128	3.5	44.9
GCC	A	40	1.1	14.3	49	1.3	20.7	35	0.9	14.4	57	1.5	20.0
GCA	A	38	1.0	13.6	51	1.4	21.5	70	1.9	28.8	73	2.0	25.6
GCG	A	53	1.4	18.9	44	1.2	18.6	37	1.0	15.2	27	0.7	9.5
GAT	D	62	1.7	82.7	64	1.7	79.0	61	1.6	74.4	47	1.3	61.8
GAC	D	13	0.4	17.3	17	0.5	21.0	21	0.6	25.6	29	0.8	38.2
GAA	E	33	0.9	36.3	42	1.1	47.2	38	1.0	45.2	51	1.4	61.4
GAG	E	58	1.6	63.7	47	1.3	52.8	46	1.2	54.8	32	0.9	38.6
GGT	G	73	2.0	24.0	103	2.8	35.2	86	2.3	31.0	55	1.5	20.4
GGC	G	25	0.7	8.2	48	1.3	16.4	36	1.0	13.0	52	1.4	19.3
GGA	G	94	2.5	30.9	58	1.6	19.8	73	2.0	26.4	84	2.3	31.1
GGG	G	112	3.0	36.8	84	2.3	28.7	82	2.2	29.6	79	2.1	29.3

[a] "Total #" refers to the absolute number of times each codon is used across the 13 protein-encoding genes of each taxon; "% total" refers to the number of times each codon is used relative to the total number of codons comprising all 13 protein-encoding genes.
[b] "% amino acid" refers to the percentage that each codon is used relative to others that code for the same amino acid.
